# Inhibition of exosome biogenesis affects cell motility in heterogeneous sub-populations of paediatric-type diffuse high-grade gliomas

**DOI:** 10.1186/s13578-023-01166-5

**Published:** 2023-11-13

**Authors:** Giulia Pericoli, Angela Galardi, Alessandro Paolini, Lucia Lisa Petrilli, Gerardo Pepe, Alessandro Palma, Marta Colletti, Roberta Ferretti, Ezio Giorda, Stefano Levi Mortera, Anna Burford, Andrea Carai, Angela Mastronuzzi, Alan Mackay, Lorenza Putignani, Chris Jones, Luisa Pascucci, Hector Peinado, Manuela Helmer-Citterich, Emmanuel de Billy, Andrea Masotti, Franco Locatelli, Angela Di Giannatale, Maria Vinci

**Affiliations:** 1 Department of Onco-hematology, Gene and Cell Therapy, Bambino Gesù Children’s Hospital-IRCCS, Rome, Italy; 2 Multifactorial and Complex Phenotype Research Area, Bambino Gesù Children’s Hospital-IRCCS, Rome, Italy; 3 Department of Biology, University of Rome “Tor Vergata”, Rome, Italy; 4 Core Facilities research laboratories, Bambino Gesù Children’s Hospital-IRCCS, Rome, Italy; 5 Multimodal Laboratory Medicine Research Area, Bambino Gesù Children’s Hospital, IRCCS, Rome, Italy; 6 Department of Molecular Pathology, The Institute of Cancer Research, Sutton, UK; 7 Oncological Neurosurgery Unit, Department of Neuroscience and Neurorehabilitation, Bambino Gesù Children’s Hospital-IRCCS, Rome, Italy; 8 Department of Veterinary Medicine, University of Perugia, Perugia, Italy; 9 Microenvironment & Metastasis Group, Molecular Oncology Program, Spanish National Cancer Research Centre (CNIO), Madrid, Spain

**Keywords:** Paediatric-type diffuse high-grade glioma, DIPG, GBM, Heterogeneity, Cell communication, Exosome, GW4869, Invasion, Migration

## Abstract

**Background:**

Paediatric-type diffuse High-Grade Gliomas (PDHGG) are highly heterogeneous tumours which include distinct cell sub-populations co-existing within the same tumour mass. We have previously shown that primary patient-derived and optical barcoded single-cell-derived clones function as interconnected networks. Here, we investigated the role of exosomes as a route for inter-clonal communication mediating PDHGG migration and invasion.

**Results:**

A comprehensive characterisation of seven optical barcoded single-cell-derived clones obtained from two patient-derived cell lines was performed. These analyses highlighted extensive intra-tumour heterogeneity in terms of genetic and transcriptional profiles between clones as well as marked phenotypic differences including distinctive motility patterns. Live single-cell tracking analysis of 3D migration and invasion assays showed that the single-cell-derived clones display a higher speed and longer travelled distance when in co-culture compared to mono-culture conditions. To determine the role of exosomes in PDHGG inter-clonal cross-talks, we isolated exosomes released by different clones and characterised them in terms of marker expression, size and concentration. We demonstrated that exosomes are actively internalized by the cells and that the inhibition of their biogenesis, using the phospholipase inhibitor GW4689, significantly reduced the cell motility in mono-culture and more prominently when the cells from the clones were in co-culture. Analysis of the exosomal miRNAs, performed with a miRNome PCR panel, identified clone-specific miRNAs and a set of miRNA target genes involved in the regulation of cell motility/invasion/migration. These genes were found differentially expressed in co-culture versus mono-culture conditions and their expression levels were significantly modulated upon inhibition of exosome biogenesis.

**Conclusions:**

In conclusion, our study highlights for the first time a key role for exosomes in the inter-clonal communication in PDHGG and suggests that interfering with the exosome biogenesis pathway may be a valuable strategy to inhibit cell motility and dissemination for these specific diseases.

**Supplementary Information:**

The online version contains supplementary material available at 10.1186/s13578-023-01166-5.

## Background

Paediatric-type diffuse high-grade gliomas (PDHGG) are a family of highly aggressive and heterogeneous tumours of the central nervous system that arise in children and young adults and for which there is still no effective treatment [1, 2]. Biologically and clinically distinct PDHGG tumour types have been defined based on their unique genetic alterations, specific anatomic locations, age at diagnosis and histopathological features [1, 3]. In the latest World Health Organization (WHO) classification of the tumours of the central nervous system (CNS), a large portion of the PDHGG family is represented by the diffuse midline glioma H3K27-altered (DMG-H3K27). This tumour type can arise in any of the midline structures including the thalamus, the pons and the spinal cord and is characterised by the K27M substitution on histones H3 and/or by EGFR or EZHIP alterations [1, 4]. Another large group of PDHGG is represented by the diffuse paediatric-type high-grade glioma histone H3 wild-type and IDH1-WT (PDHGG-WT). This tumour type typically affects the cerebral hemispheres and is less defined, in terms of molecular alterations, compared to other PDHGG. It is mainly characterised by the absence of mutations in histone H3 and IDH1 genes, the presence of oncogenic alterations, and a methylation profile more similar to low-grade tumour types [1, 3].

A significant degree of genetic and phenotypic, inter- and intra-tumour heterogeneity, at both spatial and temporal levels, has been described for PDHGG, representing one of the most challenging aspects in the effort of developing effective therapeutic strategies for these diseases [5–10]. The sub-clonal architecture of PDHGG has been demonstrated using whole genome and exome sequencing analysis of longitudinally and multi-region resected samples [7, 10]. Moreover, using primary patient-derived cell lines and multifluorescent optical barcoded-derived clones, we have shown that PDHGGs are composed of heterogeneous cell subpopulations that behave like functional networks conferring a more aggressive phenotype compared to the individual derived sub-clones [10, 11].

Intercellular communication is essential for the coordination of several functions in multicellular systems particularly important for cancer progression. This complex process mainly takes place through the active transfer of molecules from one cell to another. Cells communicate with each other via direct cell–cell contact, such as juxtracrine signalling through the gap junctions [12, 13] and tunnelling nanotubes [14, 15], as well as through indirect cell–cell communication, including autocrine and paracrine signalling [16]. It is well recognized that the communication between tumour cells and their microenvironment is essential for tumour progression and metastasis [17, 18].

In this context, exosomes, extracellular vesicles of 40–160 nm in diameter secreted by most cells, have been shown to play crucial roles in mediating intercellular communication [19, 20]. These cell membrane-derived nanovesicles contain mRNAs, miRNAs, double-stranded DNA, lipids and proteins. Upon release from their cell of origin, exosomes can be internalized by “recipient” cells through interaction with cytoplasmatic membrane receptors, plasma membrane fusion or endocytosis [19, 20]. The exosome cargo is released inside the target cells and may generate a signal activating downstream pathways and inducing phenotypic changes. When secreted by tumour cells, exosomes may act as mediators of tumorigenesis. Indeed, by carrying bioactive and oncogenic molecules, exosomes can control a variety of cellular processes in the recipient cells which significantly impact tumour progression [21].

The role of exosomes in the aggressive behaviour of PDHGG has not been explored yet. Herein, we fully characterised optical barcoded single-cell-derived clones from two PDHGG patient-derived cell lines and investigated the role of the exosomes, with a focus on the exosome-contained miRNA (exo-miRNA), in mediating the indirect cell–cell communication and in sustaining the glioma network.

## Results

### Single-cell-derived clones display different phenotypic features

To investigate the cellular and molecular mechanisms involved in inter-clonal communication in PDHGG, we generated and characterized the phenotypic features of single-cell-derived clones from patient-derived primary cell lines. To this purpose, we have generated the optical barcoded (OB) clones from multifluorescent PDHGG-WT (OPBG-GBM002) and a DMG-H3K27-altered (OPBG-DIPG002) patient-derived cell lines, using the multifluorescent marking technology as previously described [11]. The OB clones and their bulk cell lines were characterised for their phenotypic features related to cell morphology, growth, migration, invasion and adhesion.

**Fig. 1:**
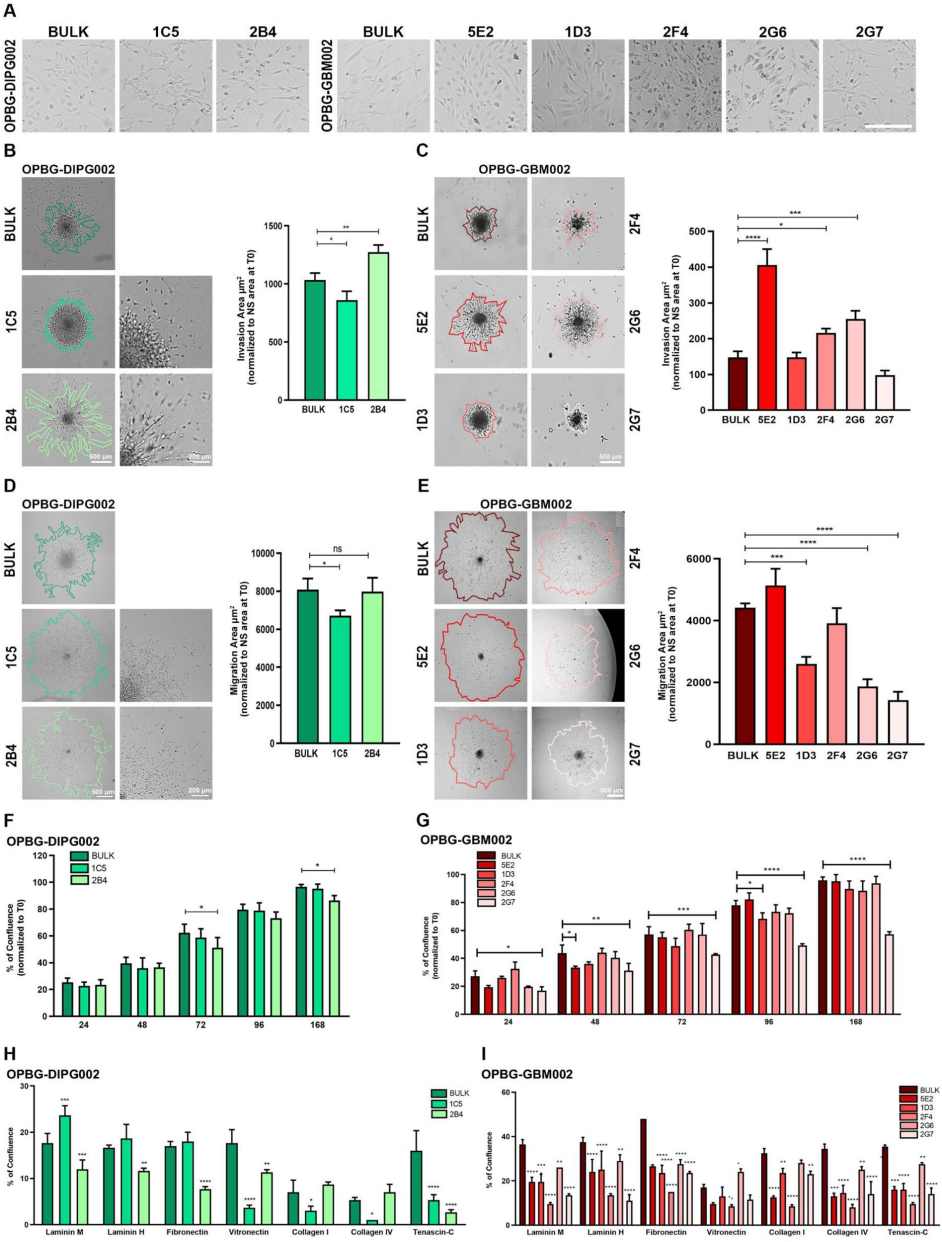
Single-cell-derived clones display different phenotypic features. **A** Representative brightfield images of the OPBG-DIPG002 and OPBG-GBM002 patient-derived and the clone cell morphologies. Scale bar = 100 μm. **B**, **C** Invasion assay was performed for 96 h with the patient-derived cells OPBG-DIPG002 (**B**) and OPBG-GBM002 (**C**) and with the respective derived clones. Brightfield images were segmented as indicated to quantify the invaded area as reported on the graph bar. Scale bar = 500 μm and 200 μm. **D**, **E** Representative images of the migration assay performed onto Matrigel for 96 h with cells of OPBG-DIPG002 (**D**), OPBG-GBM002 (**E**) and the respective derived clones. Brightfield images were segmented as indicated to quantify the migration area as reported on the graph bar. Scale bar = 500 μm and 200 μm. **F**, **G** Graph bar representing the results of the cell proliferation assay performed with OPBG-DIPG002 (**F**), OPBG-GBM002 (**G**) and the respective derived clones. After seeding, brightfield images were acquired at the indicated times and cell confluency was determined as described in the material and methods. The percentage of cell confluence corresponds to the cell confluency at the indicated time point normalised to the confluency at the time zero (t0). **H**, **I** Graph bar representing the results of the OPBG-DIPG002 (**H**), OPBG-GBM002 (**I**) and the derived clone cell adhesion assays using the 7 indicated extracellular matrices. All brightfield images were acquired and analysed with the Celigo imaging cytometer and are representative of 3 independent biological repeats. Data are mean ± SD, n = 3. (****) p < 0.0001; (***) p < 0.001; (**) p < 0.01; (*) p < 0.05

Individual clones displayed different cell morphologies (Fig. 1A), as exemplified by the clone 1C5 which had a larger and round cell shape when compared to the more elongated features of 2B4, both derived from OPBG-DIPG002. Moreover, the clone 5E2, derived from OPBG-GBM002, presented a smaller cell size compared to clones 1D3 and 2G7 which were fusiform with well-pronounced cell protrusions (Fig. 1A). Interestingly, the 2 bulk cell lines and each clone differed also in terms of invasion (Fig. 1B, C) and migration (Fig. 1D, E) ability. The cells from the OPBG-DIPG002-derived 1C5 clone presented an ameboid-like invasion and migration pattern in contrast to the more mesenchymal-like pattern observed with the cells of the 2B4 (Fig. 1B). These clones also displayed a significantly different degree of invasion and migration when compared to their bulk population (Fig. 1B, D). The OPBG-GBM002 bulk cell line and its derived clones had a very low cell invasive capability even if, the 5E2 and 2G6 showed a significantly higher degree of invasion than the bulk and the other cellular clones (Fig. 1C). On the contrary, the bulk cell population and its derived clones displayed high but heterogeneous migration capacity (Fig. 1E). The clone 5E2 demonstrated a significantly higher degree of migration when compared to the OPBG-GBM002 bulk cell line and the other 3 derived clones, with the 1D3, 2G6 and 2G7 being the least migratory.

Furthermore, the clones exhibited overall different growth rates, also when compared to the bulk cell line from which they were derived (Fig. 1F, G). The single-cell-derived clones and the bulk cell lines exhibited a heterogeneous adhesion capacity (Fig. 1H, I). Overall, the cell lines OPBG-DIPG002 and OPBG-GBM002 displayed a higher adhesion capability on all seven matrices tested compared to their respective clones. For the OPBG-DIPG002, 1C5 showed stronger adhesion property on Laminin (mouse and human), Fibronectin and Tenascin-C, while 2B4 adhered more on Vitronectin, Collagen I and IV (Fig. 1H). Furthermore, the OPBG-GBM002-derived 2G6 and 2F4 clones, displayed the highest and the lowest adhesion capacity respectively, on all the matrices tested (Fig. 1I). Therefore, the clones and the bulk cell lines exhibited a heterogeneous adhesion phenotype.

**Fig. 2:**
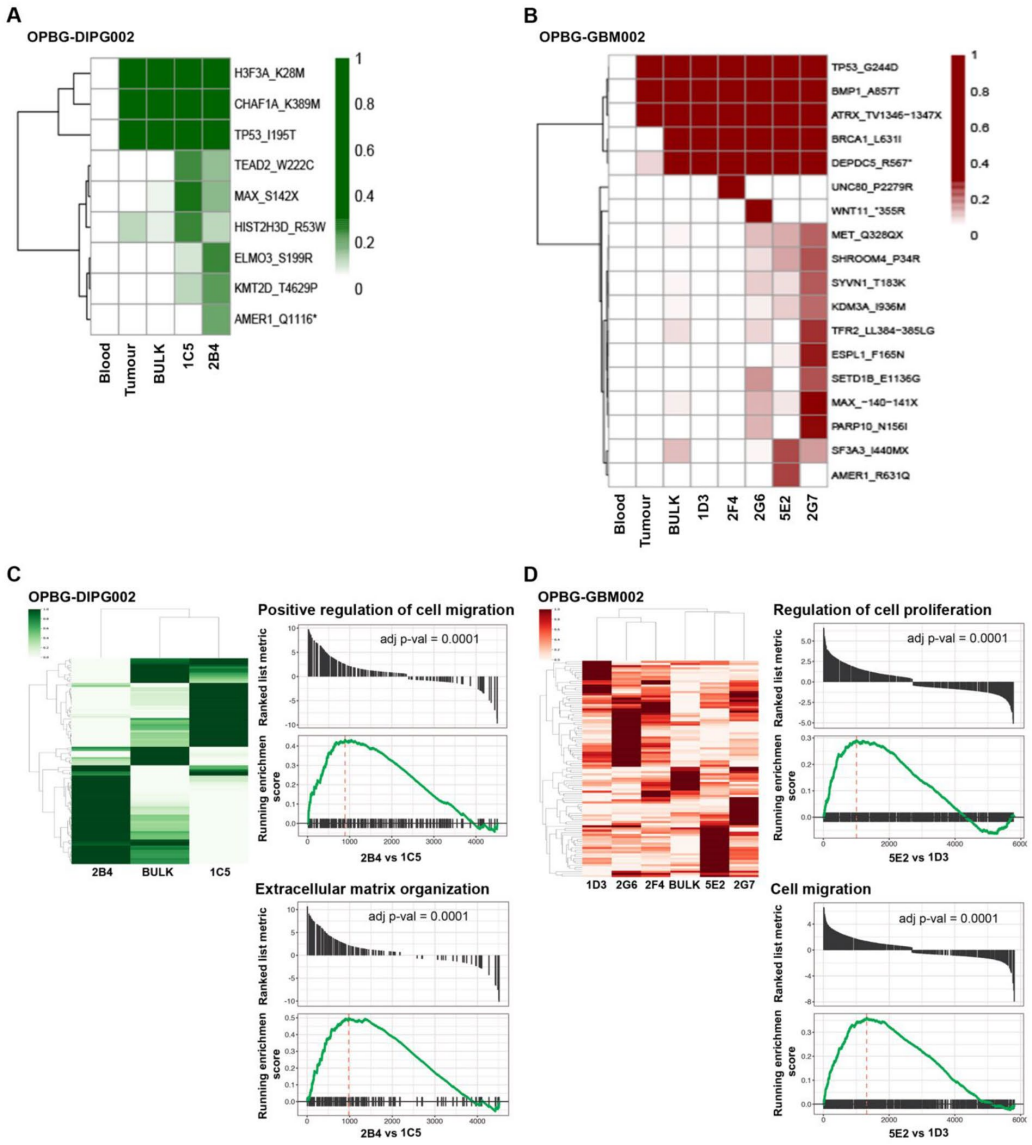
Single-cell-derived clones display different genomic and transcriptomic features. **A**, **B** Next-generation sequencing was carried out on the OPBG-DIPG002 (**A**), OPBG-GBM002 (**B**) cell lines and the respective tumour samples and corresponding derived clones. Blood samples were used as a control. The heatmaps show the gene variants identified in at least one specimen. **C**, **D** Heatmaps showing the differential gene expression obtained from RNA sequencing analysis performed with cells from OPBG-DIPG002 (**C**), OPBG-GBM002 (**D**) and from the corresponding derived clones. Gene set enrichment analysis was performed for the top 50 most highly differentially expressed genes between the OPBG-DIPG002 derived clones 2B4 and 1C5 (**C**) and the OPBG-GBM002 derived clones 5E2 and 1D3 (**D**). All cell preparations were sequenced n = 1 and statistical comparisons were made by Gene Set Enrichment Analysis

Then, we looked at the genomic and transcriptomic profiles of both OPBG-DIPG002 and OPBG-GBM002 bulk cell lines and their respective derived clones. Next Generation Sequencing (NGS) analysis was performed at high depth using a custom-designed targeted panel. In addition to the common mutations (*e.g. H3F3A, TP53, ATRX*), the clones showed several shared mutations, not identified in the bulk and in the patient tumour tissue (*e.g. MAX, KMT2D, SETD1B*), and clone-specific mutations (*e.g. AMER1, WINT11*) (Fig. 2A, B). RNAseq was performed to obtain the transcription profile of OPBG-DIPG002 and OPBG-GBM002 and their respective clones. Both bulk cell lines and the clones showed heterogeneous transcriptomic profiles. While the transcriptomic profile of the clone 1C5 was closely related to the one of the OPBG-DIPG002 cell line, a diverse profile characterised all the clones derived from the OPBG-GBM002 bulk cell line (Fig. 2C, D). Based on the interesting and distinctive phenotypic features evidenced for the clones (*e.g.* motility, proliferation), we performed gene set enrichment analysis (GSEA) for the 1C5 and 2B4 clones derived from OPBG-DIPG002 and for 5E2 and 1D3, the two most phenotypically different clones derived from OPBG-GBM002. Interestingly, for the OPBG-DIPG002, 2B4 showed enrichment in genes associated with positive regulation of cell migration and extracellular matrix organization when compared to 1C5 (Fig. 2C). For the OPBG-GBM002, 5E2 displayed enrichment in genes associated with the positive regulation of cell proliferation and cell migration when compared to 1D3 clone (Fig. 2D). Interestingly, the clones with a particular aggressive phenotype, 2B4 for OPBG-DIPG002 and 5E2 for OPBG-GBM002, are associated with unique gene signatures (Additional file 2: Table S1), which correlate with a worse overall survival in patients (Additional file 1: Fig S1).

These results confirm an intrinsic heterogeneity in PDHGG that is phenotypically displayed and retained by the distinct clones.

### Inter-clonal interactions during migration and invasion

Next, we wanted to investigate how the inter-clonal interaction affects PDHGG tumour cell motility. To this end, we used two phenotypically and transcriptionally distinct clones, 1C5 and 2B4 for the OPBG-DIPG002, and 5E2 and 1D3 for OPBG-GBM002, and compared their invasion and migration phenotypes when grown individually and in co-culture (Additional file 1: Fig S2A, D, G). When in co-culture, 1C5 and 2B4 clones displayed significantly higher migration and invasion phenotypes compared to their mono-culture condition (Additional file 1: Fig S2B, E). Similarly, 5E2 and 1D3 clones also displayed an enhanced migration phenotype in co-culture compared to their mono-culture condition (Additional file 1: Fig S2H).

**Fig. 3:**
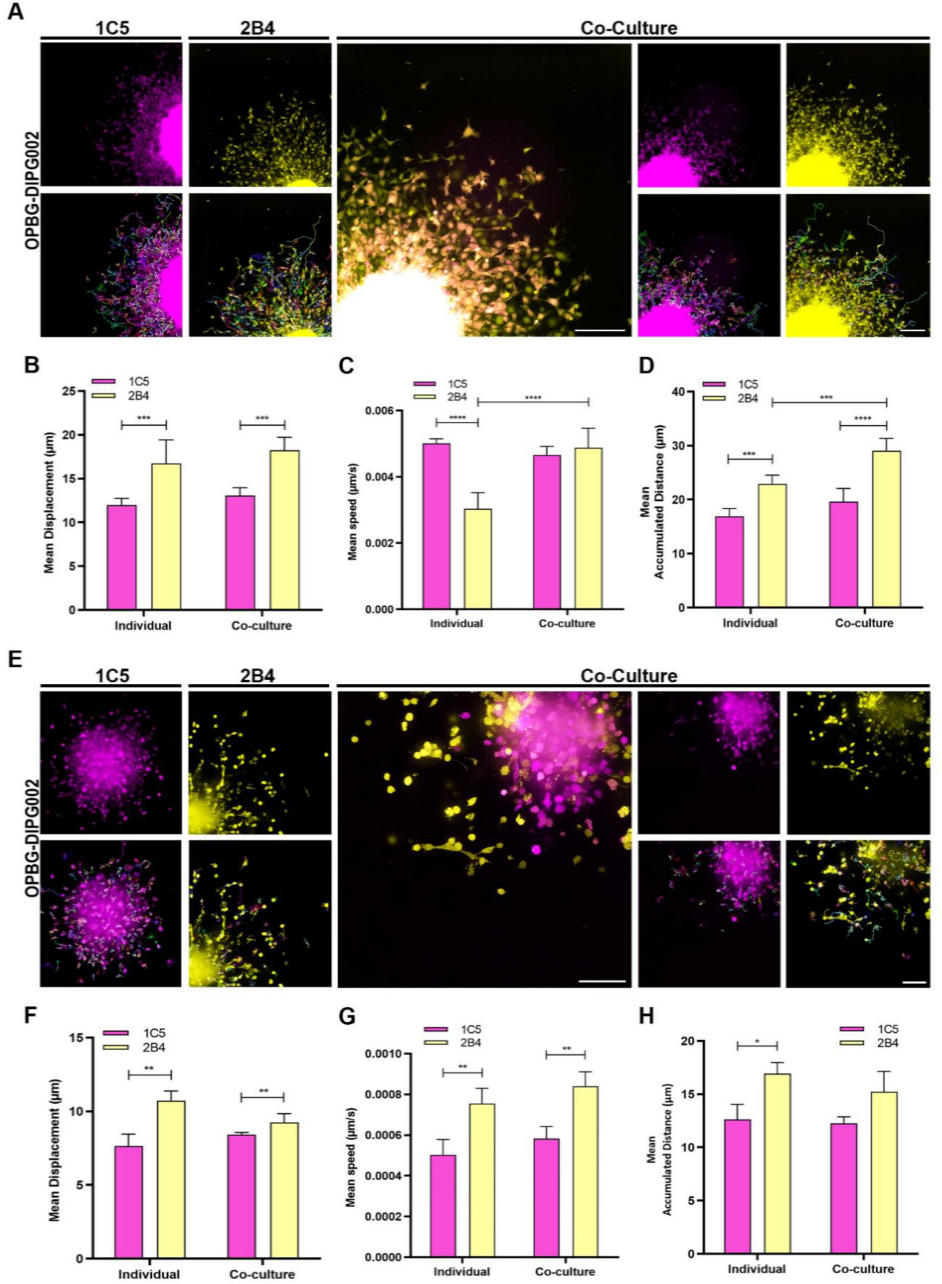
Single-cell tracking of 3D migration and invasion. Representative fluorescent images acquired with the Operetta CLS 48 h after the initiation of the 3D migration (**A**) and invasion (E) assays for OPBG-DIPG002 single-cell-derived clones 1C5 (Venus) and 2B4 (m-Orange2) either in mono or in co-culture (overlay of m-Orange2 and Venus) Scale Bar = 200 μm. For cell tracking experiments, images were acquired every 30 min with the Operetta CLS over 48 h. Single-cell tracking was performed using the Harmony software as represented with the lines and arrows overlayed on the fluorescent images and the mean cell displacement (**B**–**F**), speed (**C**–**G**) and accumulated distance (**D**–**H**) were determined as reported on the Graph bar. Data are mean ± SD, n = 3. (****) p < 0.0001; (***) p < 0.001; (**) p < 0.01; (*) p < 0.05

Taking advantage of having optical barcoded clones, we were able to image the cells with the Operetta CLS and look at the behaviour of each clone when they were in co-culture. Looking first at the percentage of cells in the invasion and migration area, we observed that the less motile clone had a higher percentage of invading and migrating cells compared to the other clone in co-culture (Additional file 1: Fig S2C, F, I). Moreover, by using the single-cell tracking on 3D migration and invasion assays, we analysed in-depth additional features of cell motility and looked at how these features were affected by the inter-clonal interaction when the clones were in co-culture (Fig. 3).

Significant differences were observed in terms of cell speed, displacement, and accumulated distance between the clones in mono- and co-culture, in migration (Fig. 3A–D) and invasion (Fig. 3E–H) assays, with the clone 2B4 (m-Orange2) being generally more “*motile*” than the 1C5 (Venus). Interestingly, 2B4 also seemed to advantage of the co-culture condition with the 1C5 clone, as it significantly showed higher speed and accumulated distance when compared to its mono-culture (Fig. 3C, D).

These results, together with our previous observation [10, 11], support our hypothesis on the role of inter-clonal interactions in contributing to more invasive and migratory phenotypes of PDHGG tumour cells.

### Primary characterization of exosomes derived from PDHGG bulk cell lines and single-cell-derived clones

**Fig. 4:**
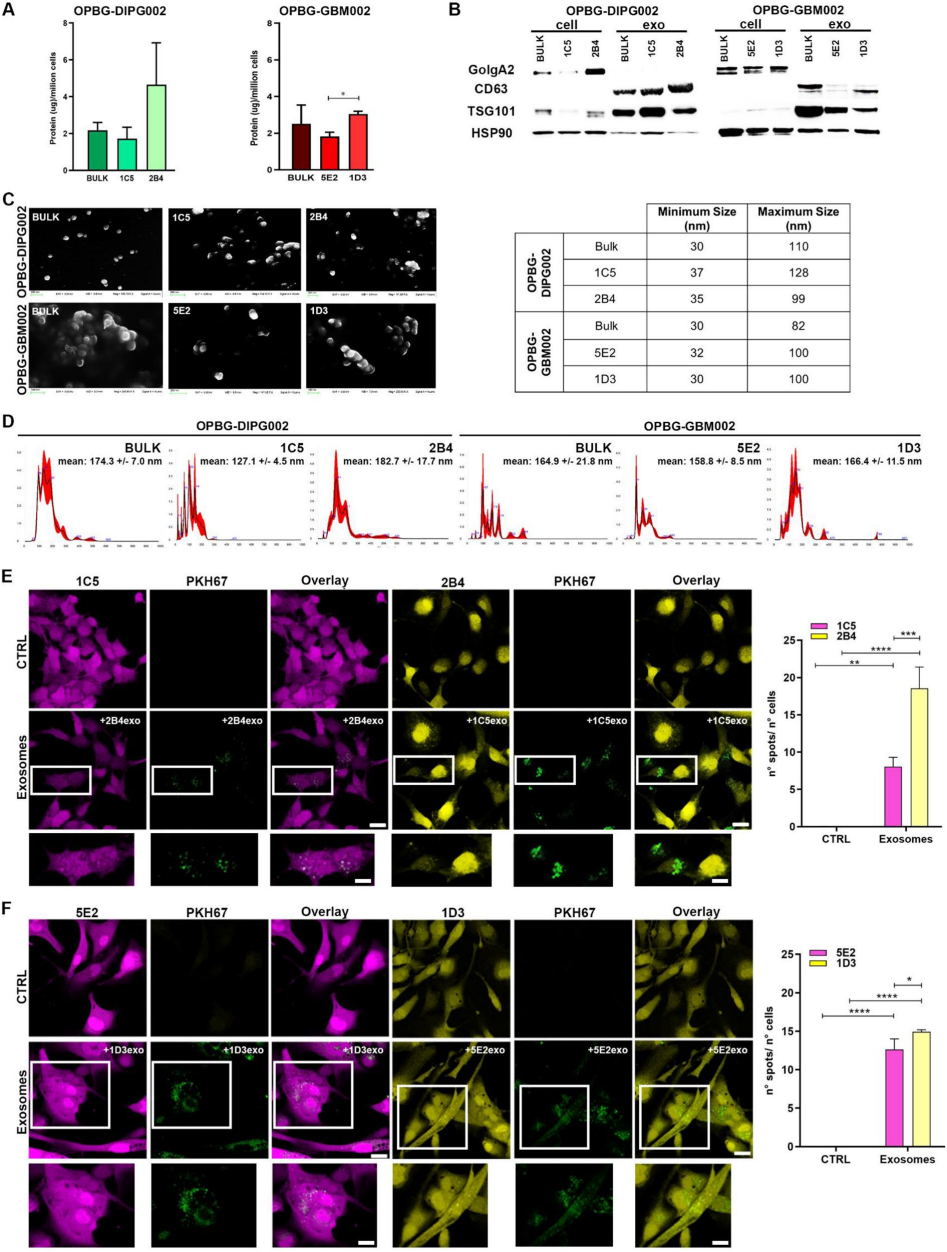
Primary characterization of exosomes and their cellular uptake. **A** Determination of the exosomal protein concentration. The quantification of the total exosomal protein obtained from 10^6^ cells of OPBG-DIPG002 and OPBG-GBM002 and of the derived clones is shown. **B** The characterization of the exosomes from the different cell lines and derived clones as in (**A**) was carried out by Western Blot for the exosomal markers, CD63 and TSG101, and non-exosomal and cell membrane marker GolgA2. HSP90 was used as the loading control. **C** Images of the Scanning Electron Microscopy show a population of heterogeneously sized exosomes isolated from the OPBG-DIPG002 and OPBG-GBM002 cell lines and the from the respective clones. Scale bar = 200 nm. The table shows the minimum and maximum size of isolated exosomes. **D** Graphics representing the size distribution of the nanoparticles resulting from the NanoSight particle-tracking analysis performed with the exosomes obtained from OPBG-DIPG002 and OPBG-GBM002 and the derived clones culture medium. **E**–**F** Representative images of the exosome uptake experiments carried out with the clones derived from OPBG-DIPG002 (**E**) and OPBG-GBM002 (**F**). The recipient clone was cultured for 24 h in the presence of 10 µg/mL PKH67-labelled exosomes isolated from the donor clone. Graph-bar represent the quantification of the PKH67 fluorescent spots by cells, corresponding to the number of PKH67-labelled exosomes internalized by cells, as determined using the Harmony software. Scale bar = 20 μm and 10 μm. Data are mean ± SD, n = 3. (****) p < 0.0001; (***) p < 0.001; (**) p < 0.01; (*) p < 0.05

We isolated and characterised extracellular vesicles (EVs) from conditioned medium (CM) of the OPBG-DIPG002 and OPBG-GBM002 bulk cell lines and their corresponding derived clones. The total EV protein content was quantified, showing a relatively variable protein amount between the bulk and the clones (Fig. 4A). Western Blot (WB) analysis demonstrated an enrichment of exosome-specific proteins, such as the tetraspanin CD63 and the tumour susceptibility gene 101 protein (TSG101) and, as expected, a decrease in Golgin subfamily A member 2 protein (GolgA2), which, instead was found in the total cell lysate (Fig. 4B). The SEM analysis, used to analyse the morphology and size of individual vesicles, with a detection limit of 0.5 nm [22], revealed single and aggregated round-shaped EVs, the majority of which ranged from 30 to 100 nm for both multifluorescent bulk cell lines and clones (Fig. 4C). The NanoSight tracking system analysis, which is used for distribution and particle concentration, with a detection limit of 60 nm [22], showed a relatively consistent EV size distribution with peaks between 100 and 150 nm (Fig. 4D).

Altogether, these results confirmed that we successfully isolated, from our cell cultures, EVs corresponding to exosomes.

### From donor to recipient clone: exosome uptake

To explore whether the exosomes secreted by the clones could exert a role in the inter-clonal interaction, we first performed exosome uptake experiments. Exosomes were isolated from CM of distinct clones: 1C5 and 2B4 derived from OPBG-DIPG002 and 5E2 and 1D3 from OPBG-GBM002. PKH67-labelled exosomes isolated from one “*donor*” clone, were added to the culture of the “*recipient*” clone, and vice versa. After 24 h, internalized fluorescent green signals were visualized in the cytoplasm of the cells (Fig. 4E, F). The quantification of the number of spots per cell showed a differential uptake between the clones. In particular, from the OPBG-DIPG002 bulk cell line, the 2B4 clone significantly internalized more 1C5-derived exosomes than the reverse (Fig. 4E). For the OPBG-GBM002 cell line, the 1D3 clone showed a higher exosome uptake compared to 5E2 (Fig. 4F).

These results demonstrate an active internalization of exosomes between “donor” and “recipient” PDHGG-derived clones.

### The inhibition of exosome biogenesis affects the single-cell-derived clone motility in mono- and co-culture conditions

Next, we wanted to explore the possibility that exosomes could play a role in regulating the motility of these cells. To address this issue, we first evaluated the effect of exosome education on cell migration and invasion. Exosomes isolated from the “*donor*” clone were used to stimulate the “*recipient*” clone, while cells were undergoing migration or invasion. At the end of the education period, we observed that neither the migration nor the invasion of the recipient clones was affected by the exosomes of the donor clones when compared to liposomes-treated control cells (Additional file 1: Fig S3A, B).

To further explore the possibility that exosomes affect the migratory/invasive capability of the clones, we used the phospholipase inhibitor GW4869 [23], a known inhibitor of exosome biogenesis.

We used the compound at doses that do not affect cell viability (10 μM for OPBG-DIPG002 and 20 μM for OPBG-GBM002 clones, respectively, Additional file 1: Fig S4) and showed that GW4869 inhibits exosome biogenesis/secretion in our clones at a variable rate, up to 65% of inhibition, with the clone 2B4 been more consistently affected (Additional file 1: Fig S5).

**Fig. 5:**
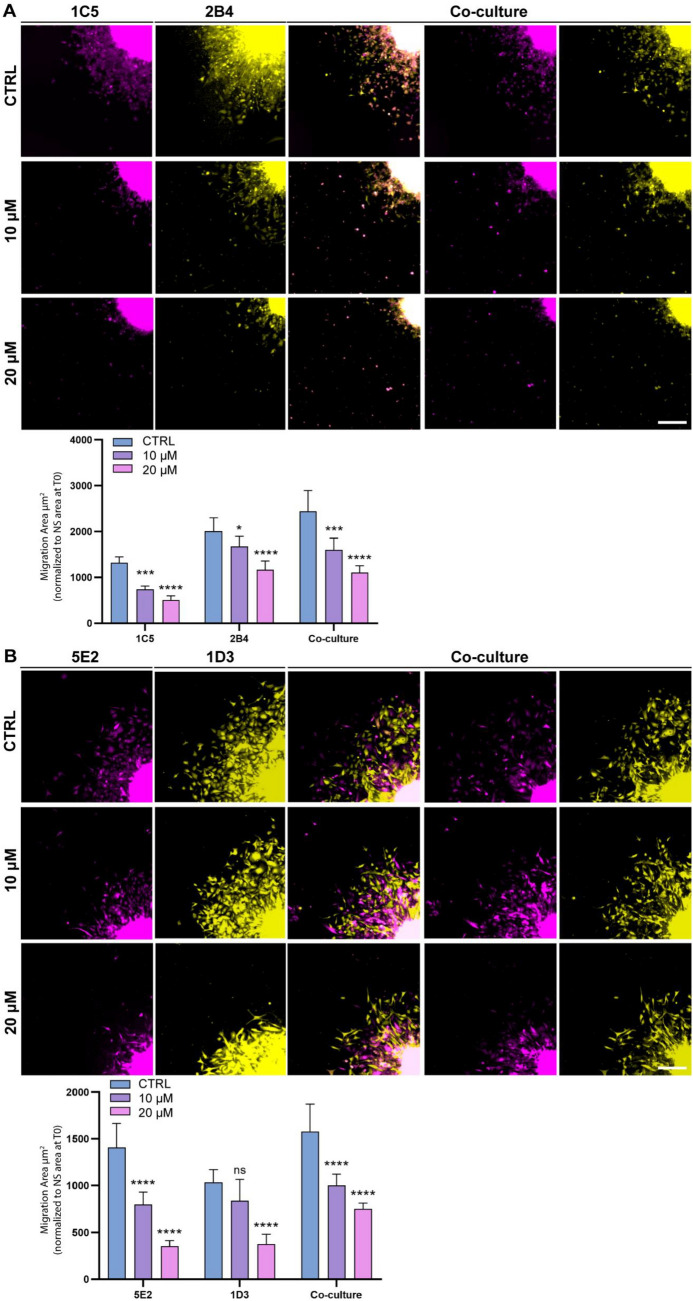
GW4869 treatment affects the migration capability of the single-cell-derived clones. **A**, **B** Representative fluorescent images of the cell migration assays performed with the OPBG-DIPG002 derived clones (**A**) 1C5 (Venus) and 2B4 (m-Orange2), and the OPBG-GBM002 clones (**B**) 5E2 (Venus) and 1D3 (m-Orange2), in mono-culture and in co-culture (overlay of m-Orange2 and Venus) and in presence or absence of 10 μM and 20 μM of GW4869 or DMSO as vehicle control for 48 h. The graph bar shows the quantification of the cell migration areas after segmentation and analysis of the images using the Harmony software. Images were acquired with the Operetta CLS every 30 min over 48 h. At the 48 h time point, Scale bar = 200 μm. Data are mean ± SD, n = 2 with 6 different technical repeats. (****) p < 0.0001; (***) p < 0.001; (**) p < 0.01; (*) p < 0.05

Based on this, we then tested the effect of GW4869 on cell motility. We performed 3D migration assays with the clones grown in mono- and co-culture condition in presence or absence of the GW4869 compound (Fig. 5). GW4869 inhibited, in a dose-dependent manner, the cell migration of the clones either in mono- or co-culture.

Furthermore, we performed single-cell tracking analysis to evaluate the effect of the inhibition of the exosome biogenesis on several parameters linked to cell motility, including accumulated distance, cell displacement and speed (Additional file 1: Fig S6A, B). For the OPBG-DIPG002 cell line, both clones, 2B4 and 1C5, appeared to be affected by the treatment with GW4869 (Additional file 1: Fig S6A). Upon treatment, cells of the clone 1C5 showed a significantly reduced displacement and speed either in mono- and co-culture conditions compared to the untreated control, while the cells of the clone 2B4 displayed a significant reduction in all the measured parameters. For the OPBG-GBM002 clones, the 5E2 and the 1D3 showed a strong reduction of the measured parameters (Additional file 1: Fig S6B).

Finally, we explored the possibility that exosomes could re-stimulate cell migration following the GW4869 treatment. To this end, exosomes from the “donor” clone were used to stimulate the migration in the “recipient” clone, in the presence or absence of GW4869. Interestingly, in the presence of GW4869, the exosomes obtained from the “donor” clone were able to rescue, even if partially, the GW4869-mediated inhibition of cell migration of the recipient clone, when compared to the relative control (Additional file 1: Fig S7).

These results indicate that exosome biogenesis plays a role in the motility of PDHGG cells and suggest that the inter-clonal communication mediated by the exosomes could contribute, at least in part, to the aggressive PDHGG cell phenotype.

### miRNA analysis of exosome cargo from single-cell-derived clones

To determine how the inhibition of the exosome biogenesis affects PDHGG cell motility, we first analysed the cargo of the exosomes secreted by the single-cell-derived clones in terms of miRNAs.

For the identification of exosomal miRNAs (exo-miRNAs), we used a 384 miRNome PCR panel. Following the analysis, we did not identify differentially expressed exo-miRNAs between the paired clones, 1C5 and 2B4 for OPBG-DIPG002 and 5E2 and 1D3 for OPBG-GBM002. However, we found that the expression of some of the exo-miRNAs was clone specific. The miR-200c-3p was found exclusively expressed in the exosomes isolated from the clone 1C5, while miR-887-3p, miR-885-5p, and miR-582-5p were found exclusively expressed in the exosomes secreted by the clone 2B4, both OPBG-DIPG002 derived clones (Table 1). For the clones derived from OPBG-GBM002, miR-203a and miR-877-5p were found exclusively expressed in the exosomes of 5E2, while miR-572, miR-376a-3p, and miR-22-3p were only expressed in the exosome isolated from 1D3 (Table 1).

**Table 1:** Exosome miRNAs in OPBG-DIPG002 and OPBG-GBM002 single-cell-derived clones

Cell line	Single-cell-derived clone	exo-miRNAs	Target gene	Protein name	References
OPBG-DIPG002	1C5	hsa-mir-200c-3p	*CRKL*	CrkL	[24]
			*FLNA*	FLNA	[25]
			*FN1*	FN	[26]
			*GLI3*	GLI3	[27]
			*NTRK2*	NTRK2	[28]
			*PTPRZ1*	PTPRZ	[29]
			*TUBB2A*	TUBB2A	[30]
			*VEGFA*	VEGF-a	[31]
			*DDIT4*	DDIT4	[32]
	2B4	hsa-mir-582-5p	*MNX1*	MNX1	[33]
			*RAC1*	RAC1	[34]
			*NR2F2*	NR2F2	[35]
			*TBX20*	TBX20	[36]
		hsa-mir-885-5p	*CTNNB1*	β-Catenin	[37]
			*PAFAH1B1*	PAFAH1B1	[38]
			*RAC1*	RAC1	[34]
			*MNX1*	MNX1	[33]
		hsa-miR-887-3p	–	–	–
OPBG-GBM002	5E2	hsa-mir-877-5p	*MAPK8*	MAPK8	[39]
			*NR2F2*	NR2F2	[35]
			*AMOTL2*	AmotL2	[40]
			*NDE1*	NudE	[41]
			*TUBB2B*	TUBB2B	[42]
		hsa-miR-203a	–	–	–
	1D3	hsa-mir-22-3p	*NTRK2*	NTRK2	[28]
			*PEX5*	PEX5	[43]
			*FUBP1*	FUSE-binding protein 1	[44]
			*DDIT4*	DDIT4	[32]
		hsa-mir-376a-3p	*FOXG1*	FOXG1	[45]
			*PDS5A*	PDS5 homolog A	[46]
			*AMOTL2*	AmotL2	[40]
		hsa-miR-572	–	–	–

Table of exclusive exo-miRNAs found in the clones and their predicted target genes involved in migration and/or invasion processes

### Identification of exosome miRNA target genes associated with migration and invasion

Given our interest in the potential role of the exosomes in migration and invasion processes, we performed further analysis to identify the target genes of the clone-specific exo-miRNAs involved in these processes. For the OPBG-DIPG002-derived clones, we identified 9 genes targeted by miR-200c-3p exclusively identified in the 1C5-derived exosomes (Table 1) and 6 genes predicted to be targets of miR-885-5p and miR-582-5p, that were specifically identified in the exosomes of the 2B4 clone (Table 1), with *MNX1 and RAC1*2 genes being common targets of the two miRNAs. For the OPBG-GBM002 derived clones, we identified 5 genes as predicted targets of miR-877-5p, found in 5E2-derived exosomes (Table 1), and 7 targets for miR-376a-3p and miR-22-3p exclusively expressed in the 1D3 exosomes (Table 1).

We identified 3 genes, *NTRK2, DDIT4 and NR2F2,* as common targets of the exo-miRNAs identified in the clones derived from both OPBG-DIPG002 and OPBG-GBM002. Interestingly, these 3 genes are involved in the regulation of cell invasion and/or migration phenotype.

### The inhibition of exosome biogenesis modulates the expression of genes regulating migration/invasion in single-cell-derived clones

We hypothesized that the inhibition of exosome biogenesis in PDHGG clones, and hence the consequent disruption of inter-clonal communication via exosomes, could affect their motility capability (Fig. 5A, B).

**Fig. 6:**
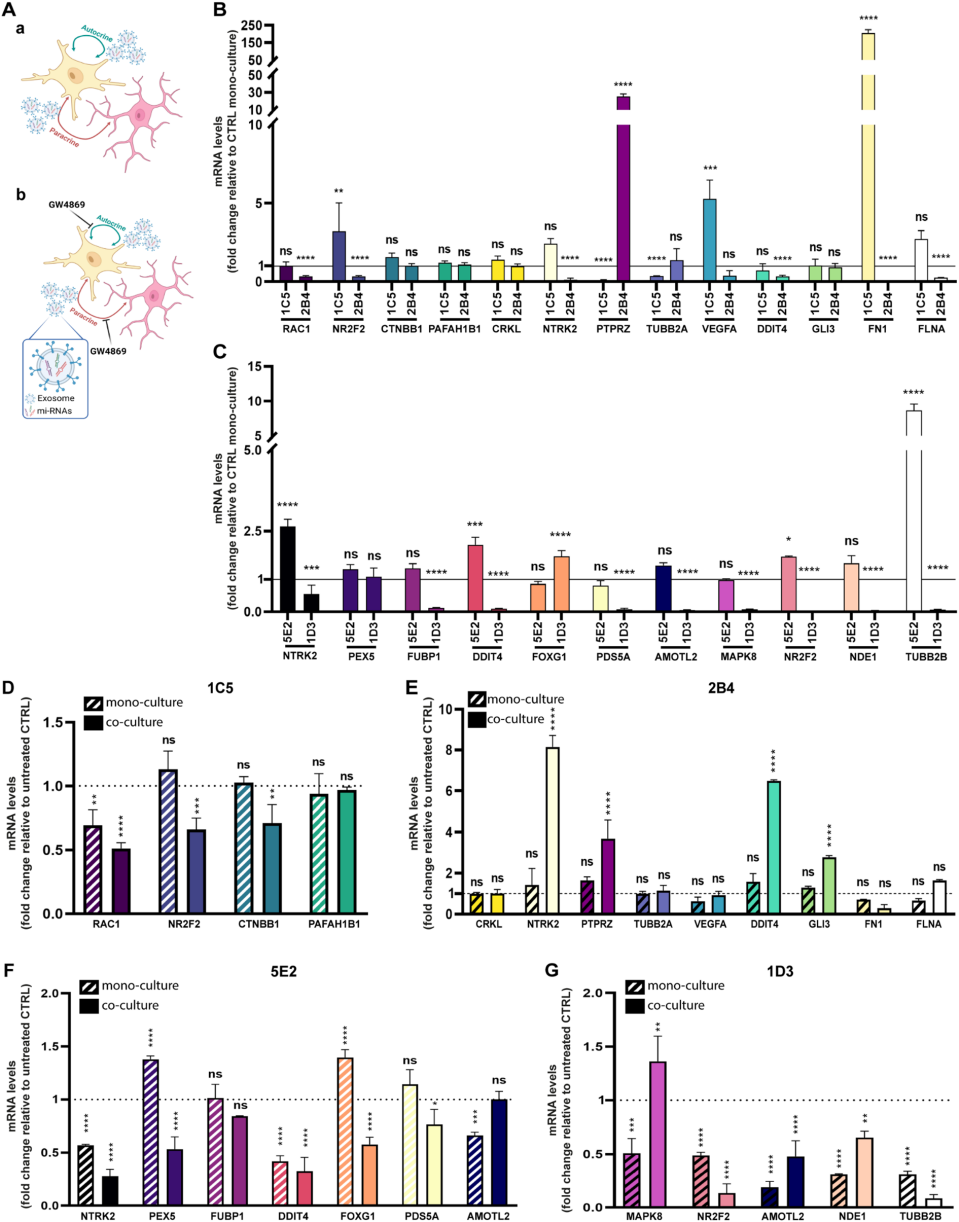
The inhibition of exosome biogenesis modulates the expression of migratory/invasive related genes in single-cell-derived clones. **A** Scheme representing the hypothetical mode of action of GW4869. Single-cell-derived clones in co-cultures were treated with the vehicle control (a) or with the GW4869 (b) for 96 h. **B**, **C** The graph-bar shows the fold change in mRNA expression between a clone grown in mono and co-culture conditions. The mRNA levels were analysed by Q-PCR for the indicated and selected set of genes involved in migration and invasion phenotypes and for the clones 1C5 and 2B4 (**B**) derived from OPBG-DIPG002 and the clones 5E2 and 1D3 derived from OPBG-GBM002 (**C**). **D**, **E** Graph-bars represent the fold change in mRNA expression of the indicated genes, analysed by Q-PCR between cells treated and not treated with 10 μM of GW4869 for the OPBG-DIPG002 derived clones 1C5 (**D**) and 2B4 (**E**) grown for 96 h in mono- (dash-bars) or in co-culture (plain coloured-bar) conditions. **F**, **G** Same as D and E but with the clones 5E2 (**F**) and 1D3 (**G**) derived from OPBG-GBM002 and treated with 20 μM of GW4869. For the gene see Table 1. Data are mean ± SD, n = 3. (****) p < 0.0001; (***) p < 0.001; (**) p < 0.01; (*) p < 0.05

To test this hypothesis, we first studied the expression levels of the exo-miRNA target genes regulating migration and invasion discussed above, for the individual clones in mono and in co-culture (Table 1 and Fig. 6A a, B and C). When in co-culture, and in comparison to the mono-culture, we observed an overall modulation of the gene expression for both 1C5 and 2B4 clones derived from OPBG-DIPG002 (Fig. 6B), as well as for the clones 5E2 and 1D3 derived from OPBG-GBM002 (Fig. 6C). In the co-culture condition, out of the 15 genes identified as targets of the exo-miRNAs of OPBG-DIPG002 derived clones (Table 1), 4 of them, *NR2F2*, *TUBB2A*, *VEGFA* and *FN1* were upregulated in 1C5, and 8 were found either downregulated, *RAC1, NR2F2, NTRK2, DDIT4, FN1* and *FLNA,* or upregulated, *PTPRZ1*, in 2B4 (Fig. 6B). Two of the 15 target genes, *MNX1* and *TBX20*, were not detected in any of the conditions analysed. For the OPBG-GBM002 derived clones, out of the 11 genes targeted by the identified exo-miRNA (Table 1), 9, including *NTRK2, FUBP1, DDIT4, PDS5A, AMOTL2, MAPK8, NRF2, NDE1* and *TUBB2* were found down-regulated and one *FOXG1* upregulated in 1D3, while 4, *NTRK2, DDIT4, NR2F2* and *TUBB2B*, were found upregulated in 5E2 (Fig. 6C). Interestingly, the mRNA expression levels of *NTRK2, DDIT4 and NR2F2*, identified as common targets of the exo-miRNAs, were found modulated in the clones 2B4, 1D3 and 5E2, except for the clone 1C5, for which only the NR2F2 mRNA expression level was found modulated. Furthermore, proteomic analysis performed on the clones from mono and co-culture conditions, demonstrated the expression and the modulation at protein level, of several miRNA predicted target genes (Additional file 1: Fig S8 and Additional file 3: Table S2).

These data suggest that, when the clones are in co-culture, the modulation of the specific exo-miRNAs targeted genes is the result of an inter-clonal cross-talk.

We have demonstrated that the inhibition of the exosome biogenesis affects the cell motility of PDHGG clones (Fig. 5). Moreover, we have identified exo-miRNA target genes involved in processes such as cell migration and invasion (Table 1). Therefore, to confirm the role of the exosomes in inter-clonal communication and, in particular, in the modulation of genes relevant to PDHGG inter-clonal motility, we repeated the mono- and co-culture experiments in the presence or absence of the exosome biogenesis inhibitor, GW4869 (Fig. 6A a and b). After 96 h of GW4869 treatment, we analysed, in each clone, the mRNA expression levels of the specific genes targeted by the exo-miRNAs produced by the “*brother*” clone (Table 1). For the OPBG-DIPG002 derived clones, a decrease in *RAC1, NR2F2* and *CTBNN1* mRNA expression levels was observed in the cells of the clone 1C5 in the co-culture condition (Fig. 6D) and, on the other hand, a significant increase in the mRNA levels of *NTRK2, PTPRZ1, DDIT4* and *GLI3* in the cells of 2B4, was detected when two clones were in co-cultures (Fig. 6E) and in comparison to their respective untreated control. Regarding the OPBG-GBM002 cell line, 5E2 was characterised by a significant modulation of the mRNA expression levels of *NTRK2, PEX5, DDIT4* and *FOXG1* upon GW4869 treatment either in mono- and co-culture conditions (Fig. 6F). The 1D3 clone also showed a modulation of gene expression. In particular, we observed a significant increase in *MAPK8* expression when 1D3 was in co-culture with 5E2, while there was an overall significant decrease of the *NR2F2, AMOTL2, NDE1* and *TUBB2B* expression in both mono and co-culture conditions (Fig. 6G).

Altogether, these results strongly support the involvement of the exosomes in the inter-clonal regulation of the mRNA expression of genes implicated in PDHGG cell migration and invasion through the transport of clone-specific miRNAs.

## Discussion

The genetic and phenotypic inter- and intra-tumoral heterogeneity may be one of the most challenging obstacles in the development of effective therapies for cancer [47–49]. Bulk DNA sequencing, single-cell RNAseq and more recently single-cell proteomic analyses have provided evidence of the intra-tumour heterogeneity in PDHGG [10, 50, 51]. Moreover, diffusely infiltrating gliomas are heterogeneous tumours organized into functional cellular networks as demonstrated in vitro and in vivo using patient-derived cell lines and orthotopic xenograft models [10, 52, 53]. These functional cellular networks are facilitated by different routes of cell–cell communications taking place between heterogeneous tumour cell populations and between tumour cells and neurons as well as other elements of the tumour microenvironment [52, 54]. The elucidation of mechanisms underlying such cell–cell interactions could lead to the identification of new therapeutic approaches to treat these devastating cancers.

In this study, we have first demonstrated multiple levels of intra-tumour heterogeneity in two different PDHGG cell lines, one PDHGG-WT and one DMG-H3K27, and investigated the role of exosomes as one of the indirect cellular crosstalk mediating PDHGG migration and invasion. We have shown that genomic and phenotypic intra-tumoural heterogeneity is retained in patient-derived cell lines stably transduced for the generation of OB single-cell derived clones [11]. The cells from different OB clones displayed marked phenotypic differences in terms of morphology, growth, invasion, migration, and adhesion. The DMG-H3K27 derived clones not only demonstrated a different invasive and migratory capability, but also a different cellular motility pattern. Interestingly, the clone 1C5 showed an ameboid-like invasion pattern, similar to the phenotype observed with the bulk cell line it was derived from, while 2B4 was characterised by a mesenchymal-like phenotype.

We have previously shown that PDHGG-derived clones interact with each other and that this interaction is key in conferring an aggressive phenotype [10]. More recently, Haider et al. used an in silico spatial computational modelling in association with in vitro co-culture experiments and were able to classify and quantify the inter-clonal interactions associated with invasive DMG cells [55]. Here, to further dissect the nature and the effect of PDHGG inter-clonal interaction taking place during cell motility, we take advantage of the OB associated to single-cell-derived clones [11] and focus our investigations on clones genetically and phenotypically different, derived from PDHGG-WT and DMG-H3K27 multifluorescent bulk cell lines. By live single-cell tracking analysis, we demonstrated that, when the single-cell-derived clones are in co-culture, they increase their speed and travelled distance compared to when they are in monoculture. It is interesting to note that for both cell lines, the clones that display higher migration and invasion capacity did not show a growth advantage over the other clones but were present in a smaller cell fraction when in co-culture. These results are in line with our previous reports [10, 11] and confirm that inter-clonal cell–cell interaction permits the acquisition of a more pronounced migratory/invasive phenotype and can be driven by less dominant/proliferative clones. The role of the interplay between clones in maintaining tumour aggressiveness has been previously demonstrated in ovarian and breast cancers. In particular, a commensal mechanism of clonal cooperation promoting metastasis has been identified in ovarian tumours [56]. In breast cancers, it has been demonstrated that cellular subclones, via secreted factors, extracellular vesicles, and physical interactions, can increase aggressiveness in other clones and, at the same time, contribute to tumour progression and metastasis [57].

Then, we questioned how different PDHGG cellular clones communicate with each other and hypothesised that one of the indirect routes may be mediated by exosomes. Secreted by most cell types and abundantly by tumour cells, exosomes are emerging as mediators of tumorigenesis and the secretion of exosomes is known to be hijacked and dysregulated in cancer [21, 58]. The existence and the roles of exosomes have been well-established in adult GBM. In GBM cells, exosomes mediate the transfer of histones, miRNA, and oncogenic molecules such as EGFRvIII [59–61]. In addition, exosomes from GBM cells may modify cell surface protein expression and cytokine secretion as well as influence the immunity functions of the tumour microenvironment, suggesting a role in the context of intra-tumour heterogeneity [62]. Recently, Tűzesi et al. demonstrated that paediatric glioma stem cells release exosomes and their miRNA content profile differs from that of normal neural stem cell exosomes. Moreover, glioma stem cells -exosomes influence the gene expression of normal neural stem cells, targeting genes involved in cell fate and tumorigenesis [63].

However, exosome-mediated intercellular communication among PDHGG heterogeneous subpopulations has not been investigated yet. Here, we show that PDHGG multifluorescent cell lines and their OB clones secrete extracellular vesicles that, based on the size and marker expression, can be defined as exosomes. Then, we demonstrate that these exosomes are actively internalized by cells and can be exchanged between “*donor*” and “*recipient*” clones. Next, we tested the hypothesis of whether they can be involved in the inter-clonal crosstalk mediating PDHGG cell migration and invasion phenotypes. We first verified that the exosomes obtained from the *donor* clones were able to affect the migration and/or invasion capability of the *recipient* clones. Surprisingly, within the timeframe and exosome concentration tested, we did not observe a response to the exogenous exosome stimuli. This may be explained by the presence in the culture medium of growth factors and supplements that represent themselves as strong stimuli that may have masked a potential response to the exosomes from the donor clones. In addition, the effect of the exogenous paracrine signalling from a donor clone could have been masked by the exosome autocrine signalling coming from the recipient clone. Both autocrine and paracrine mechanisms of exosome-mediated cell–cell communication have been demonstrated. For instance, exosome actin-associated protein cytosolic gelsolin transforms chemo-sensitive ovarian cancer cells into resistant counterparts through both autocrine and paracrine mechanisms [64]. Moreover, Tang et al. have recently shown how exosomes regulate both tumour and stromal cell migration via both autocrine and paracrine mechanisms [65].

Despite the lack of phenotype on migration and invasion induced by exosome from *donor* to *recipient* clones, we decided to further explore our hypothesis on the role played by the exosomes in mediating PDHGG migration/invasion phenotype, by testing the effect of the inhibition of the exosome biogenesis/secretion. To achieve this, we used GW4869, a symmetrical dihydroimidazolo-amide compound, which is a specific and potent inhibitor of the membrane neutral sphingomyelinase (nSMase). SMase converts sphingomyelin into ceramide, a rigid lipid element essential for the generation and release of exosomes. GW4869 has been used in different studies that proved its activity in blocking the exosome-mediated transfer of key regulators of oncogenic processes [66–69]. Here, we demonstrated that GW4869 inhibits the secretion of exosomes in our single-cell-derived clones and, as a result, significantly decreases the cell motility, including reduction of migrated area and of different parameters linked to cell motility, such as speed, displacement, and distance. These effects were observed in a dose-dependent manner, at sub-cytotoxic doses of compound, and in some cases, these effects were more pronounced when the clones were in co-culture than when in monoculture. Despite the clear effect of GW4869 treatment on the decrease in PDHGG cell motility, we did not observe a complete inhibition of the migratory capability of the single-cell-derived clones. This may be due to the existence of other routes involved in exosome biogenesis/secretion, which may not be affected by GW4869 treatment. In fact, there are at least two main pathways leading to exosome biogenesis and secretion. The endosomal sorting complexes required for transport machinery (ESCRT)-independent pathway, affected by GW4869 treatment, and the ESCRT-dependent pathway, which is sensitive to the treatment of other small molecules such as Manumycin A [23]. In light of this consideration, a combination of these two inhibitors could exert a stronger inhibition on tumour cell motility because of a more efficacious inhibition of the exosome biogenesis/secretion machinery. Alternatively, other inhibitors such Tipifarnib and Ketoconazole, could be tested given the recent evidence for their activity on exosome biogenesis through both the ESCRT-dependent and independent pathways [70]. Moreover, besides the exosomes, other mechanisms of cell–cell communication are also known to contribute to and affect tumour cell motility. These include mechanisms, such as the secretion of signalling molecules and direct mechanisms via gap junctions and tunnelling nanotubes [71–74].

As mentioned above, miRNAs are among the key effectors of exosome transfer, and we decided to focus on these molecules to study their impact on inter-clonal communication and cell motility. We characterised the exosome miRNome from two phenotypically different clones derived from two PDHGG cell lines. While from the analysis performed, we did not obtain differentially expressed exo-miRNAs between the clones analysed, we discovered several exo-miRNAs that were exclusively expressed in individual clones. In the DMG-H3K27 cell line, the mir-200c-3p was found in the exosomes isolated from the clone 1C5, while the miR-887-3p, miR-885-5p, and miR-582-5p were detected in 2B4-derived exosomes. The miR200-c is particularly of interest for its implication in regulating glioma cell growth and invasion. Its overexpression impaired glioma cell proliferation and invasion by targeting myosin and preventing the invasion and migration of adult GBM cells [75, 76]. Furthermore, miR-885-5p and miR-582-5p were detected in adult glioma cells in which they are respectively involved in the inhibition of invasion [77] and in the improvement of stem cell survival [78]. With regards to the PDHGG-WT cell line, we found miR-203a and miR-877-5p in the exosomes isolated from the clone 5E2, while miR-572, miR-376a-3p, and miR-22-3p were exclusively identified in the exosomes isolated from the 1D3 clone. It has been demonstrated that miR-877-5p, miR-376a-3p and miR-22-3p are involved in the regulation of glioma cell proliferation, dissemination, and resistance to treatments [79–81]. These results demonstrate that the single-cell-derived clones co-existing within the same tumour have different exosomal miRNA cargos providing novel evidence of the intra-tumour heterogeneity in PDHGG. Exo-miRNAs, having multiple functions and potentially different fates, may be linked to the specific metabolism and/or phenotype of the cells they are produced from, or on the contrary, may be expelled in the extracellular space to get simply rid of them [82–84].

Given our interest oi the inter-clonal signals mediating migration and invasion, we interrogated miRNA databases to identify the exo-miRNA target genes involved in the regulation of cell motility. Interestingly, we identified several target genes involved in the modulation of migration, invasion, and metastasis. Among these miRNA-predicted targeted genes, it is worth mentioning *RAC1, PTPRZ, TUBB2A VEGFA, FN1, FLNA* for OPBG-DIPG002 derived-clones, and *FUBP1, FOXG1, PDS5A, AMOTL2, MAPK8, NDE1*, *TUBB2B* for OPBG-GBM002 derived-clones, for which we demonstrated a significant modulation of their expression levels when the clones were in co-culture compared to the mono-culture condition. Three additional genes known to be involved in PDHGG cell motility processes*, NTRK2, DDIT4* and *NRF2* [28, 32, 35] were also found differentially expressed in the co-culture when compared to the mono-culture condition. Interestingly, these 3 genes are common targets of exo-miRNAs identified in both OPBG-DIPG002 and OPBG-GBM002 derived clones, suggesting that their expression and modulation through the exosome signalling could be key in the regulation of the cell motility phenotype. Furthermore, we found that the expression level of several proteins related to genes targeted by these miRNAs was modulated between the mono- and co-culture conditions.

Our data are in line with several evidence showing that cell–cell communication can modulate gene expression in interconnected cellular systems [85]. We demonstrated that the inhibition of the exosome biogenesis via GW4689 induces a significant modulation of the expression level of genes targeted by exo-miRNA when the clones are in co-culture more than in mono-culture condition. Altogether, our work provides for the first time evidence that part of the inter-clonal communication occurring between heterogeneous populations of PDHGG is mediated via the exosome machinery. Interfering with the exosome biogenesis/secretion machinery not only inhibits cell motility but also affects the expression of key exo-miRNA target genes involved in migration/invasion processes. This indicates that exosomes contribute to the inter-cellular signalling involved in the regulation of the cell migration/invasion phenotype of PDHGG. Lastly, the exosomes are known to be secreted by neurons and are implicated in the modulation of synaptic plasticity [86]. Glioma-neuronal synaptic activity is an emerging hallmark in glioma progression [87, 88]. Expanding on the role of exosomes in the communication between glioma cells and neurons may offer additional opportunities to inhibit the glioma intercellular connectivity [53] and cell dissemination.

## Conclusions

In summary, we demonstrate, for the first time, that PDHGG cells can secrete, internalize and exchange exosomes between different cellular clones. Moreover, we show that the miRNA content of these exosomes is clone specific highlighting the heterogeneity of the processes implicated in the exosome-mediated inter-clonal communication. Exosomes represent a vehicle involved in the crosstalk between PDHGG heterogeneous cell populations and our results support that their secretion contributes, at least in part, to the cell dissemination of this disease.

Further investigations will be necessary to fully elucidate the mechanisms by which exosomes mediate communication between heterogeneous cell populations in PDHGG. How exosomes are internalized and which other signalling molecules are implicated in the exosome-mediated crosstalk between PDHGG cells, are questions that remain to be elucidated. Understanding these mechanisms will offer novel opportunities to interfere with the inter-clonal crosstalk in PDHGG to weaken these aggressive cancers.

## Materials and methods

### Cell cultures

PDHGG patient-derived cell lines were established either immediately, after collection (biopsy or resection) of fresh tissues, or from live cryopreserved tissues as previously described [10, 11]. The hemispheric PDHGG-WT cell line, OPBG-GBM002, and the DMG-H3K27, OPBG-DIPG002, were cultured adherent on laminin (Merck) in serum-free, tumour stem-cell media (TSM). Briefly, as previously described [10, 11], the medium composition is: 1:1 Neurobasal(-A) (Invitrogen), and DMEM: F12 (Invitrogen), supplemented with Anti-mycotic/Anti-biotic, HEPES, NEAA, GlutamaX, Sodium Pyruvate (Invitrogen) and B27(-A) (Invitrogen), human bFGF (20 ng/mL), human EGF (20 ng/mL), human PDGF-AA (10 ng/mL) and PDGF-BB (10 ng/mL) (Peprotech) and heparin (2 ng/mL) (Stem Cell Technologies). The cell authenticity was verified using short tandem repeat (STR) DNA fingerprinting by Eurofins Genomics. Cell cultures were routinely tested and verified mycoplasma-free. The study was conducted according to the guidelines of the Declaration of Helsinki and approved by the Institutional Ethical Committee of the Bambino Gesù Children’s Hospital (Ethical Committee Approvals N°1680/2018). Informed consent was obtained from all subjects involved in the study.

### High-throughput phenotypic analysis

To perform the cell proliferation assay, cell suspensions (final concentration is 10^3^ cells/well) were dispensed into laminin pre-coated 96-well flat-bottom plates (view-plates, PerkinElmer, Waltham, MA, USA). Starting from time zero (T = 0), automated image analysis was carried out on a CeligoS cytometer (PerkinElmer, Waltham, MA, USA) at intervals of 24 h until the end of the experiment (7 days), using the Confluence application. The data were plotted as the percentage of the total area in the field of view covered by cells (% of confluence) normalised to the time zero (n = 3).

3D invasion assays were performed as previously described [11, 89], with some modifications. Briefly, for neurosphere (NS) generation, 100 μl/well of cell suspensions at optimized densities were dispensed into ultra-low attachment (ULA) 96-well round-bottom plates (Corning, New York, NY, USA) and, when the neurospheres reached a size of 300–350 μm in diameter, the invasion assay was performed. Plates were placed on ice and after removing 50 μl of medium from each well, 50 μl of Matrigel were gently dispensed/well and plates were incubated at 37 °C, 5% CO_2_. Starting from time zero (intended as the beginning of the invasion assay, upon the embedding of the NS in the Matrigel), and every 24 h for a total of 4 days, automated image analysis was carried out on the CeligoS cytometer using the cell Confluence application. The degree of cell invasion in the Matrigel was evaluated and the data were plotted as the invaded area normalised to the area of NS at time zero (n = 3).

3D migration assays were performed as previously described [11, 90] with some modifications. When the NS reached a size of 250–300 μm in diameter, the migration assay was performed. Briefly, flat-bottom 96-well plates View Plate (PerkinElmer) were coated for 2 h at RT with 125 μg/ml Matrigel (Corning) in the culture medium in the absence of growth factors. Once the coating was completed, a total of 200 μl/well of culture medium was added to each well. A total of 50 μl of medium was removed from ULA 96-well round-bottom plates containing NS, and the remaining medium including the NS was transferred into individual wells of Matrigel pre-coated 96 flat-bottom plates. Starting from time zero (intended as the beginning of the migration assay, upon the transfer of the NS onto the Matrigel) and at intervals up to 24 h for 4 days, automated image analysis was carried out on the Celigo cytometer as described for the invasion assay. The degree of cell migration was evaluated, and the data were plotted as the migrated area normalised to the area of the NS at time zero (n = 3).

The adhesion assay was performed as previously described [91], with some modifications. The flat-bottom 96-well plates View Plates (PerkinElmer) were coated for 2 h at 37 °C with mouse Laminin (Merck), Human Laminin (Merck), Vitronectin (Merck), Tenascin-C (Merck), Collagen I (Merck), Collagen IV (Merck) at 10 μg/ml in PBS with Ca^2+^/Mg^2+^ or HBSS, and for 2 h at RT with Fibronectin (Merck) at 10 μg/ml in PBS with Ca^2+^/Mg^2+^. To detach the adherent cells, they were incubated with a non-enzymatic dissociation buffer consisting of PBS with 2–5 mM ethylenediaminetetraacetic acid (EDTA), for 15–20 min at RT and then centrifuged at 1300 rpm for 5 min at RT. The cell suspension was diluted in an integrin-binding buffer (1:1 HBSS and PBS with Ca^2+/^Mg^2+^, 0.1% BSA, 25 mM HEPES and dispensed at a final concentration of 10^5^ cells/well/100 μl). The plate was incubated for 1 h at 37 °C. Subsequently, the wells were washed twice with 200 μl of PBS, discarding the PBS by turning the plate and shaking it, and automated images were acquired and analysed with the Celigo cytometer using the Confluence application. The degree of cell adhesion was quantified, and data were plotted as the percentage of the total area covered by adherent cells in the field of view (% of confluence) (n = 3).

For co-culture experiments, cells from single-cell-derived clones, 5E2 and 1D3 derived from OPBG-GBM002 patient-derived cell line and 1C5 and 2B4 derived from OPBG-DIPG002 patient-derived cell line, were seeded at a cell ratio of 50:50. Migration and invasion assays as well as automated image analysis were carried out as described above (n = 3). In addition, to quantify the contribution of each clone to the specific cellular process (migration and invasion), automated image acquisition was performed using the Operetta CLS (PerkinElmer, Waltham, MA, USA) and image analysis was performed with the Harmony software to quantify the percentage of every clone based on their specific optical barcodes [11] (n = 3).

Live imaging experiments for single-cell tracking, and automated fluorescent image acquisition were performed as previously described [11] with the Operetta CLS (PerkinElmer, Waltham, MA, USA) every 30 min for 96 time points, starting from 24 h after the beginning of the migration and invasion assays. To clearly distinguish the two clones, based on their optical barcodes [11], the m-Orange and Venus fluorescent signals were respectively acquired for OPBG-DIPG002 2B4 and OPBG-DIPG002 1C5. The Harmony software (PerkinElmer) on the Operetta was used to evaluate cell speed, accumulated distance and displacement, and calculate the overall invasion and migration area (n = 5). Two independent experiments were performed.

### DNA extraction and targeted sequencing

DNA was extracted using DNeasy Blood & Tissue Kit (Qiagen) according to the manufacturer’s instructions. DNA from primary tumours, blood, bulk multifluorescent cell lines and single-cell-derived clones were subjected to targeted sequencing using a panel of 333 genes recurrently mutated in PDHGG [10]. Targeted sequences were aligned to hg19 with bwa, and variants were called using GATK v2.3.9 best practices. Variants were annotated with the ensemble variant effect predictor for consequence and heatmaps of variant allele fractions were drawn in R v4.2.1.

### RNAseq pre-processing and analysis

Raw RNA-seq data, formatted as FASTQ files, were processed following a three-step pipeline. In the first step, the adapters were removed using two different tools. Cutadapt [92] was used to remove forward (“AGATCGGAAGAGCACA CGTCTGAACTCCAGTCA”) and reverse (“AGATCGGAAGAGCGTCGTGTAGGGAAAGAGTGT”) specific adapters, subsequently TrimmomaticPE [93] was used to remove ILLUMINA specific adapters and thus sequences with a length less than 30 nucleotides were eliminated. In the second step hisat2 [94] was used to map reads to the reference genome (Human, GRCh38). Finally, the numbers of reads mapped to individual reference transcripts were counted using the Htseq package [95], which generate a tab-delimited table of reading counts for each transcript. Transcripts were sorted according to their expression variance across the samples using a Python script developed in our lab; then the first 100 transcripts with high variance in their expression profile were selected for sample clustering and heatmap representation. Samples clustering was performed using clustermap function from the seaborn python library (https://joss.theoj.org/papers/10.21105/joss.03021). Gene set enrichment analysis has been performed using clusterprofiler [96] and path-view [97] R packages. P-values for statistical significance are reported on the GSEA plots.

A gene signature was defined as the set of genes with TPM expression > 100 in one clone and < 10 in the other clones (2B4 and 1C5 for OPBG-DIPG002 and 5E2 and 1D3 for OPBG-GBM002), while simultaneously being TPM expression > 10 in the bulk population (Additional file 2: Table S1). We derived overall survival curves through GEPIA 2 web server for these highlighted genes [98]. The expression data of high-grade glioma cancer patients were downloaded from the TCGA Data Portal (https://tcga-data.nci.nih.gov) using the recommended GDC data transfer tool. The processed data (level 3) were used. Overall survival probability curves were plotted using the Kaplan- Meier method and comparisons between the curves were analysed using the log-rank test [99]. All tests were performed at the 0.05 level of significance. The samples were split into 2 groups based on the expression of 2B4 OPBG-DIPG002-derived clone or 5E2 OPBG-GBM002-derived clone specific gene signature, respectively.

### Exosome purification, characterization, and labelling

Exosomes were purified from TSM cell-conditioned medium (CM) after 5–6 days of culture when cells had reached 80–90% of confluence, in the presence or absence of GW4869. The CM was centrifuged at 500 g for 10 min to remove floating, detached cells, followed by two other centrifugations at 3000 g for 20 min and 12,000 g for 20 min to remove any possible apoptotic bodies and large cell debris. Finally, the exosomes were isolated by the ultracentrifugation method, spinning the CM at 100,000 g for 70 min twice using a 70Ti rotor (Beckman Coulter). Exosomal protein concentration was measured by bicinchoninic acid assay (BCA) (Pierce, Thermo Fisher Scientific). Exosome preparations were verified by Western Blot (WB) and Scanning Electron Microscopy (SEM) as previously described based on MISEV guidelines [100, 101]. For the WB, membranes were hybridized overnight at 4 °C with: anti-GolgA2 11308-1-AP (Proteintech), anti-CD63 (MX-49.129.5) sc-5275 (Santa Cruz Biotechnology), anti-TSG101 (4A10) ab83 (Abcam), anti-HSP90 (F-8) sc-13119 (Santa Cruz Biotechnology). The exosome size distribution and concentration were determined as previously described [100, 102] on a NS500 nanoparticle characterization system (NanoSight) equipped with a blue laser (405 nm).

The uptake experiments were performed as previously described [69] with some modifications. The exosomes and the synthetic Plain Liposomes (Cellsome, Encapsula NanoSciences) pellets were resuspended in 1 ml of mix constituted by 1 ml diluent solution C and 1 μl of fluorescent green dye (PKH67, Sigma Aldrich, PKH67 GL). After mixing at RT for 5 min, exosomes were ultra-centrifugated, resuspended in 100 μl of PBS and quantified by BCA (Pierce, Thermo Fisher Scientific). The labelled exosomes and liposomes were used at a concentration of 10 μg/ml and added to the cells plated at 70–80% of confluence. After 24 h of incubation, media was removed, and cells were washed twice with PBS and fixed with 4% PFA for 15 min. Automated fluorescent image analysis was performed at the Operetta CLS (PerkinElmer, Waltham, MA, USA) in three independent experiments (n = 3). Higher resolution images were acquired at Leica TCS AOBS-SP8X confocal microscope with 63X magnification and processed using Adobe Photoshop CS4 software (Adobe Systems Inc.).

### Exosome miRNA profile analysis

For miRNA analysis, exosomes were isolated using the miRCURY Exosome Cell/Urine/CSF Kit (Qiagen) according to the manufacturer’s indication. Briefly, 2 ml of Precipitation Buffer B was added to the CM (2.5–3 ml), collected as mentioned above, and the solution was mixed thoroughly. The sample was incubated for 60 min at 4 °C and, after that, was centrifuged at 3200 g for 30 min at 20 °C. The supernatant was removed, and the exosome pellet was resuspended in 100 μl Resuspension Buffer by vortexing for 15 s.

RNA were extracted from exosomes using Plasma/Serum Circulating and Exosomal RNA Purification Mini Kit (Slurry Format) (Norgen) following the manufacturer’s instruction. The quantification and quality evaluation was performed by using the Agilent 2100 Bioanalyzer RNA Pico assay (Agilent technologies) following the manufacturer’s instructions. Total RNA from exosomes was mixed with artificial RNAs (RNA spike-ins) used as controls, and the mixture was reverse transcribed at 42 °C for 60 min using the miRCURY LNA™ Universal RT cDNA Synthesis Kit (Qiagen) following the manufacturer’s instruction. The expression of each miRNA (including spike-ins) was evaluated by miRNome PCR Panel (384-wells, Qiagen) by quantitative PCR (q-PCR) (QuantStudio^™^ 12 K Flex Real-Time PCR System, Thermo Fisher Scientific). The amplification curves were imported into the GenEX qPCR analysis software (ver.5, Exiqon) and normalised by global mean. The mean of individual Cq values was considered to calculate the expression level (fold change [FC]).

miRNA target genes have been retrieved from the Mouse Genome Informatics database by querying for the GO term “cell migration”. The datasets have been filtered for sub-terms related to neuronal and glial cell migration and metastasis, and genes mapped to their Human orthologues. The resulting gene list has been further filtered to retain only genes that are known targets of selected miRNA, using “mirtarbase” as a reference (https://mirtarbase.cuhk.edu.cn/~miRTarBase/miRTarBase_2022/php/index.php) (for the complete list of miRNA target genes see Additional file). Networks have been drafted using Cytoscape [103].

### Single-cell-derived clone exosome education

Exosome education of PDHGG clones was performed by adding 10 µg/ml of purified clone-derived exosomes or liposomes to invading and migrating cells every 48 h for 7 days. Starting from time zero and at intervals up to 24 h for 7 days, automated image analysis was carried out on the Celigo cytometer as described above. The degree of cell migration and invasion was evaluated, and the data were plotted as a percentage of time zero (n = 3). Where indicated, the assay was performed in the presence or absence of GW4869 (see below for treatment details).

### GW4869 treatments

For the dose–response and cell viability assays, 500 cells were seeded in ULA 96-well plates (Corning), and 48–72 h post seeding, the cells were treated with GW4869 (Sigma) starting from 100 µM top concentration and in a serial dilution manner (1:3). Ninety-six hours later, the cell viability was evaluated using the CellTiter-Glo Luminescent Cell Viability Assay (Promega, WI, USA) for which luminescent counts were quantified on a Sinergy H1 microplate reader (BioTek). Data were normalised to the median signal from 0, 2% DMSO-containing wells (negative CTRL) to estimate the % of viable cells.

To evaluate the effect of GW4869 treatment on 3D migration, the assays were performed as described above with some modifications. Upon the preparation of the Matrigel pre-coated migration plate, 200 μl of the medium, containing GW4869 (10 and 20 μM), or vehicle (0, 2% DMSO), were dispensed in each well of 6–10 replicates/condition. The NS of the mono-culture and co-culture clone conditions were transferred onto the migration plates. Starting 24 h after the migration assay, automated fluorescent image acquisition was performed at the Operetta CLS (PerkinElmer) every 30 min for 96 time points. The Harmony software (PerkinElmer) was used to evaluate the cell speed, accumulated distance and displacement, as well as the migration area. Two biological repeats were performed.

To analyse the effect of GW4869 treatment on the miRNA target gene expression levels, clones in mono- and co-culture conditions were treated with the vehicle control (0.2% DMSO) or GW4869 at the indicated concentrations. After 96 h of treatment, each clone in co-culture was sorted using FACSAria III (BD bioscience) based on their specific optical barcodes: Venus for 1C5 and m-Orange for 2B4, both clones derived from OPBG-DIPG002; Venus for 5E2 and m-Orange for 1D3, both clones derived from OPBG-GBM002. After that, the cells were washed twice with PBS and centrifuged at 1300 rpm for 5 min. Cell pellets were used for RNA extraction and subsequent analysis.

### RNA-extraction, RT-PCR, and q-PCR for gene expression analysis

Total RNA for gene expression analysis was extracted using RNeasy Plus Mini Kit (Qiagen, Netherlands) according to the manufacturer’s protocol. Reverse transcription (RT-PCR) was performed using the SuperScript VILO cDNA Synthesis Kit (Applied Biosystems, CA, USA) according to the specific guidelines. Quantitative real-time PCR (q-PCR) was performed using TaqMan Fast Universal PCR Master Mix (Applied Biosystems, CA, USA) on the Applied Biosystems QuantStudio 7 K Flex Real-Time PCR Systems with the following TaqMan Gene Expression Assay primers (Applied Biosystems): *RAC1* (Hs01902432_s1); *NR2F2* (Hs00819630_m1); *CTNNB1* (Hs00355045_m1); *PAFAH1B1* (Hs00181182_m1); *CRKL* (Hs00178304_m1); *NTRK2* (Hs00178811_m1); *PTPRZ1* (Hs00960146_m1); *TUBB2A* (Hs00742533_s1); *VEGFA* (Hs00900055_m1); *DDIT4* (Hs01111686_g1); *GLI3* (Hs00609233_m1); *FN1* (Hs01549976_m1); *FLNA* (Hs00924645_m1); *PEX5* (Hs00165604_m1); *FUBP1* (Hs00900762_m1); *FOXG1* (Hs01850784_s1); *PDS5A* (Hs00374857_m1); *AMOTL2* (Hs01048101_m1); *MAPK8* (Hs01548508_m1); *NDE1* (Hs00214339_m1); *TUBB2B* (Hs00603550_g1). Each sample was normalised according to the glyceraldehyde-3-phosphate dehydrogenase (GAPDH) mRNA (Hs02786624_g1) expression level and the fold change was calculated using the 2^−ΔΔCt^ method.

### Protein extraction and digestion and liquid chromatography/tandem mass spectrometry (LC–MS/MS) analysis

After 96 h of co-culture between clones, cells were FACS sorted based on their OB. Cell pellets were lysed and proteins were extracted and quantified as previously described [104].

After lysis, 50 µg of proteins were digested with Sequencing grade Trypsin (Promega) by filter-aided sample preparation (FASP) protocol [Ref]. Obtained tryptic peptides (2 µg) were separated by liquid chromatography (HPLC) using an Ultimate 3000 RSLCnano System device (Thermofisher Scientific). A 90 min gradient, ramping from 5 to 25% aqueous solution of ACN with 0.1% formic acid, was used to elute peptides onto a PepMap RSLC C18 column (Thermo Fisher Scientific – 2 µm particle size, 100 Å porosity, 75 µm ID, 50 cm length) working at a flow rate of 250 nL/min. Peptides were eluted in the EasySpray source of an Orbitrap Fusion mass spectrometer (Thermo Scientific) and analysed by a data-dependent acquisition (DDA) method. The Orbitrap analyzer was used for full-MS scans and MS2 scans using 120000 and 15000 resolutions respectively (250–1500 m/z scan range). Fragments were obtained by high energy collision-induced dissociation (HCD) on precursor ions setting the dynamic exclusion at 30 s after a single scan. Two technical replicates from 3 biological replicates were acquired for each sample.

Protein identification and Label-Free Quantitation (LFQ) analysis were performed with the Proteome Discoverer 2.5 software (Thermo Fisher Scientific), using the SwissProt database restricted to Homo Sapiens (2022_1). Two miss cleavages were allowed, cysteine carbamidomethylation and oxidation of methionine were set as fixed and variable modifications respectively. Mass error tolerance was set to 10 ppm for precursor ions and 0.02 Da for fragment ions. The INFERYS rescoring was applied before the Percolator algorithm for false discovery rate (FDR) calculation with a cut-off of 0.01. All proteins identified with less than two peptides or no unique peptides were filtered out. Quantification was performed by determining the protein intensity ratio between mono-culture vs co-culture conditions and using the summed abundance of both unique and razor peptides, normalizing on the total peptide amount. The protein ratio was calculated with the “pairwise ratio-based” algorithm using a t-test. Up- or down-regulated proteins were selected by setting the log2 fold change value and the p-value thresholds to 0.66 and 0.05 respectively (Additional file 3: Table S2).

### Statistical analysis

Data are reported as mean ± standard deviation (SD) and statistical analysis was performed with GraphPad Prism 8.4 software. For the phenotypic characterization, statistical significance was evaluated using One-way ANOVA multiple comparisons, while for 3D live imaging experiments, statistical significance was evaluated with Two-way ANOVA Multiple Comparison Tests and Paired t-tests. For exosome uptake and exosome education experiments, statistical significance was evaluated with the Two-way ANOVA Multiple Comparison Test. For q-PCR, statistical analyses were performed on 2^−ΔΔCt^. All experiments were performed in triplicate. Throughout the study ****P < 0.0001; ***P < 0.001; **P < 0.01; *P < 0.05; ns: not significant.

### Supplementary Information

**Additional file 1: Figure S1.** Overall Survival analysis of HGG patients using TCGA gene expression data. (A-B) The patient cohort was divided into two groups based on the median expression of OPBG-DIPG002 2B4 (A) and OPBG-GBM002 5E2 (B) specific gene signatures. The red curve represents patients with higher expression of this signature, while the blue curve represents patients with lower expression. **Figure S2.** Motility of single-cell-derived clones in mono and co-culture condition. Individual clones derived from OPBG-DIPG002 (A-D) and OPBG-GBM002 (G) were cultured either alone or co-cultured at an equal cell/cell ratio. Representative brightfield images are shown for the invasion (A) and migration (D-G) assays after 96 hours. The extent of invasion and migration is marked with coloured segmentation: co-culture (light blue); mono-cultures of 1C5 and 5E2 (fuchsia); mono-cultures of 2B4 and 1D3 (yellow). The total area covered by invading and migrating cells was quantified and normalised by the NS area determined at the time t0 (B-E-H). Scale bar = 500μm. Representative fluorescent images of OPBG-DIPG002 single-cell-derived clones 1C5 (Venus) and 2B4 (m-Orange2) 3D invasion (A) and migration (D) and of OPBG-GBM002 single-cell-derived clones 5E2 (Venus) and 1D3 (m-Orange2) migration (G) are shown as mono-culture and as co-culture (overlay of Venus and m-Orange2). Fluorescent images were acquired on a Leica TCS AOBS-SP8X confocal microscope. The percentage of each clone in the invasion (C) and migration (F-I) at 96 hours was analysed with Harmony software. Data are mean ± SD, n = 3. (****) p<0.0001; (***) p<0.001; (**) p<0.01; (*) p<0.05. **Figure S3.** The exosomal education of single-cell-derived clones does not affect their migratory/invasive phenotype. Exosomal education of the clones was performed. Exosomes isolated from donor clones were used to educate the recipient clone (10 μg/mL of media) every day, over 7 days. The effect on migration (A left panel and B) and invasion (A right panel) was quantified as the total area in the field of view covered by migrating and invading cells was normalized to the size of the NS at t0. Automated image acquisition was performed on a Celigo imaging cytometer. Scale bar = 500μm. Data were derived, and representative images were obtained from n=3 independent experiments. Data are mean ± SD, n = 3. **Figure S4.** Effect of the inhibition of exosome biogenesis on cell viability. (A) Effect of exosome biogenesis inhibition by GW4869 on the viability of the cells for the clones derived from OPBG-DIPG002 (left panel) and OPBG-GBM002 (right panel) cell lines. Cells were treated with GW4869 or vehicle control. Cell viability was measured with CellTiter Glo assay and dose-response curves are shown. Results are mean ± SD; n = 3. **Figure S5.** Analysis of exosome biogenesis inhibition upon GW4869 treatment on single-cell-derived clones. (A and C) Plots showing the size distribution of the nanoparticles resulting from the NanoSight particle-tracking analysis performed with the exosomes obtained from OPBG-DIPG002 and OPBG-GBM002 derived-clones CM in the presence or absence of 10μM and 20μM of GW4869 respectively or DMSO as vehicle control (representative experiment is shown, n=2). (B and D) Determination of the exosomal protein concentration. The quantification of the total exosomal protein obtained from OPBG-DIPG002 and OPBG-GBM002 derived-clones in untreated and treated with GW4869 conditions, is shown (n=3). (E) Determination of the exosomal RNA concentration. The quantification of total exosomal RNA obtained from OPBG-DIPG002 2B4 clone, in the presence or absence of 10μM of GW4869. **Figure S6.** Effect of the inhibition of exosome biogenesis on migration of single-cell-derived clones. (A and B) The mean accumulated distance, mean displacement, and speed of OPBG-DIPG002 (A) and OPBG-GBM002 (B) clone migrating cells in mono- and co-culture are shown. Cells were treated with 10μM and 20μM GW4869 or vehicle control, for 96 hours. Analysis was performed with Harmony software. Data are mean ± SD, n = 3. (****) p<0.0001; (***) p<0.001; (**) p<0.01; (*) p<0.05. **Figure S7.** Effect of the combination GW4869+exosome treatment on migration of single-cell-derived clones. OPBG-DIPG002 1C5 and 2B4 single-cell-derived clones were treated with GW4869 (10μM) or vehicle control (CTRL), in the presence or absence of exosomes (10 μg/mL of media) isolated from donor clones. The extent of migration is marked with coloured segmentation (white). The effect on migration (A) was quantified as the total area in the field of view covered by migrating cells, normalized to the size of the NS at T0. Automated image acquisition was performed on a Celigo imaging cytometer. Scale bar = 200μm. Data were derived, and representative images were obtained from n=2 independent experiments. Data are mean ± SD. (****) p<0.0001; (***) p<0.001; (**) p<0.01; (*) p<0.05. **Figure S8.** Proteomic analysis of single-cell-derived clones. Volcano plot of the mono-culture/co-culture ratio values measured in the label-free quantitative analysis performed on single-cell-derived clones of OPBG-DIPG002 (A and B) and OPBG-GBM002 (C and D). The red and green boxes include significant up- and down-regulated proteins respectively, based on the thresholds set for abundance ratio and p-value. Identified proteins related to the miRNA-predicted target genes are highlighted with sky-blue dots and tagged with the UniProt entry name.

**Additional file 2: **Gene signatures associated with OPBG-DIPG002 2B4 derived-clone and OPBGGBM0025E2 derived-clone.

**Additional file 3: ** Summary of the proteomic Label-Free Quantitation (LFQ) analysis performed for theclones 2B4, 5E2, 1C5, and 1D3 based on the comparison of mono versus coculture conditions.

## Data Availability

All the data supporting the findings of this study are available from the corresponding author MV upon reasonable request.

## Abbreviations

PDHGG: Paediatric-type diffuse high-grade gliomas
WHO: World Health Organization
DMG-H3K27: Diffuse midline glioma H3K27-altered
PDHGG-WT: High-grade glioma histone H3 wild-type and IDH1-WT
exo-miRNA: Exosome-contained miRNA
OB: Optical barcoded
NGS: Next generation sequencing
GSEA: Gene set enrichment analysis
EV: Extracellular vesicles
CM: Conditioned medium
TSG101: Tumour susceptibility gene 101 protein
GolgA2: Golgin subfamily A member 2 protein
nSMase: Membrane neutral sphingomyelinase
STR: Short tandem repeat
NS: Neurosphere
ULA: Ultra-low attachment
BCA: Bicinchoninic acid assay
WB: Western blot
SEM: Scanning electron microscopy
FC: Fold change
RT-PCR: Reverse transcription PCR
q-PCR: Quantitative real-time PCR
GAPDH: Glyceraldehyde-3-phosphate dehydrogenase
SD: Standard deviation
Ns: Not significant
