# Supplementary material for: Inhibition of exosome biogenesis affects cell motility in heterogeneous sub-populations of paediatric-type diffuse high-grade gliomas
Source: Cell Biosci. 2023 Nov 13;13:207. doi: 10.1186/s13578-023-01166-5 (PMC10641969; doi:10.1186/s13578-023-01166-5)

Supplementary figure 1

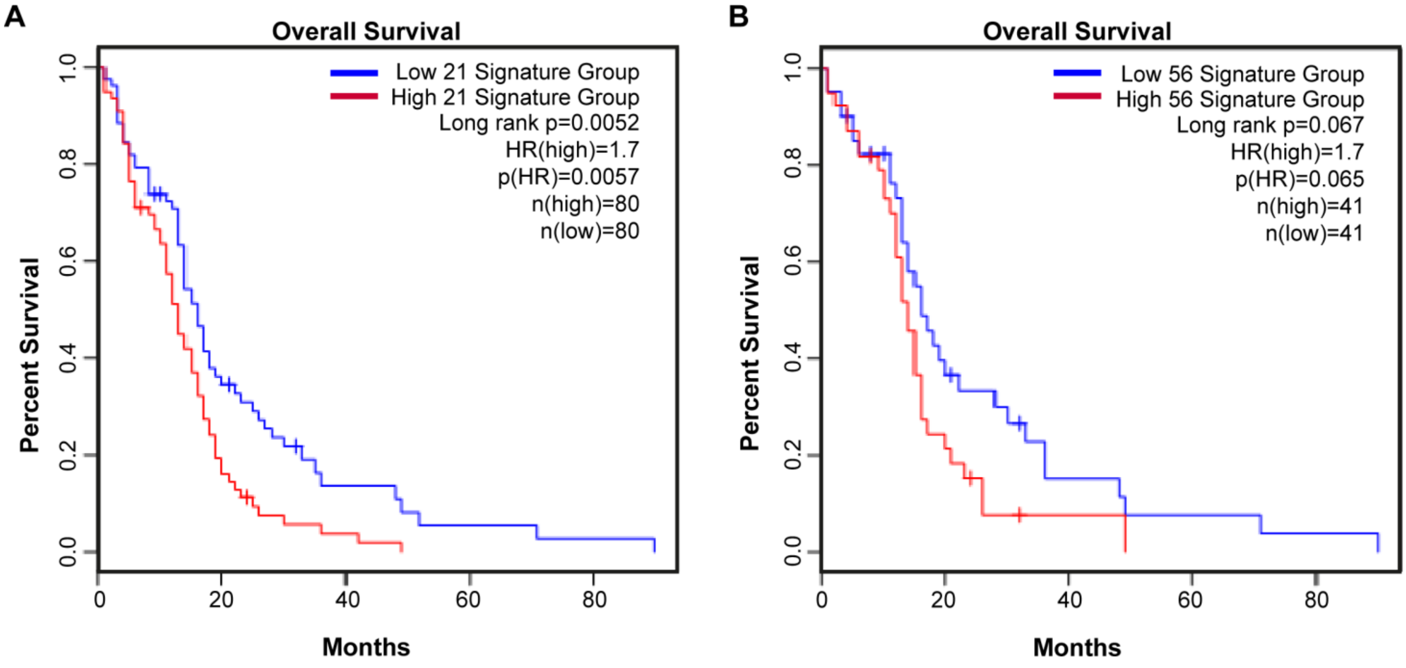

Supplemetary figure 2

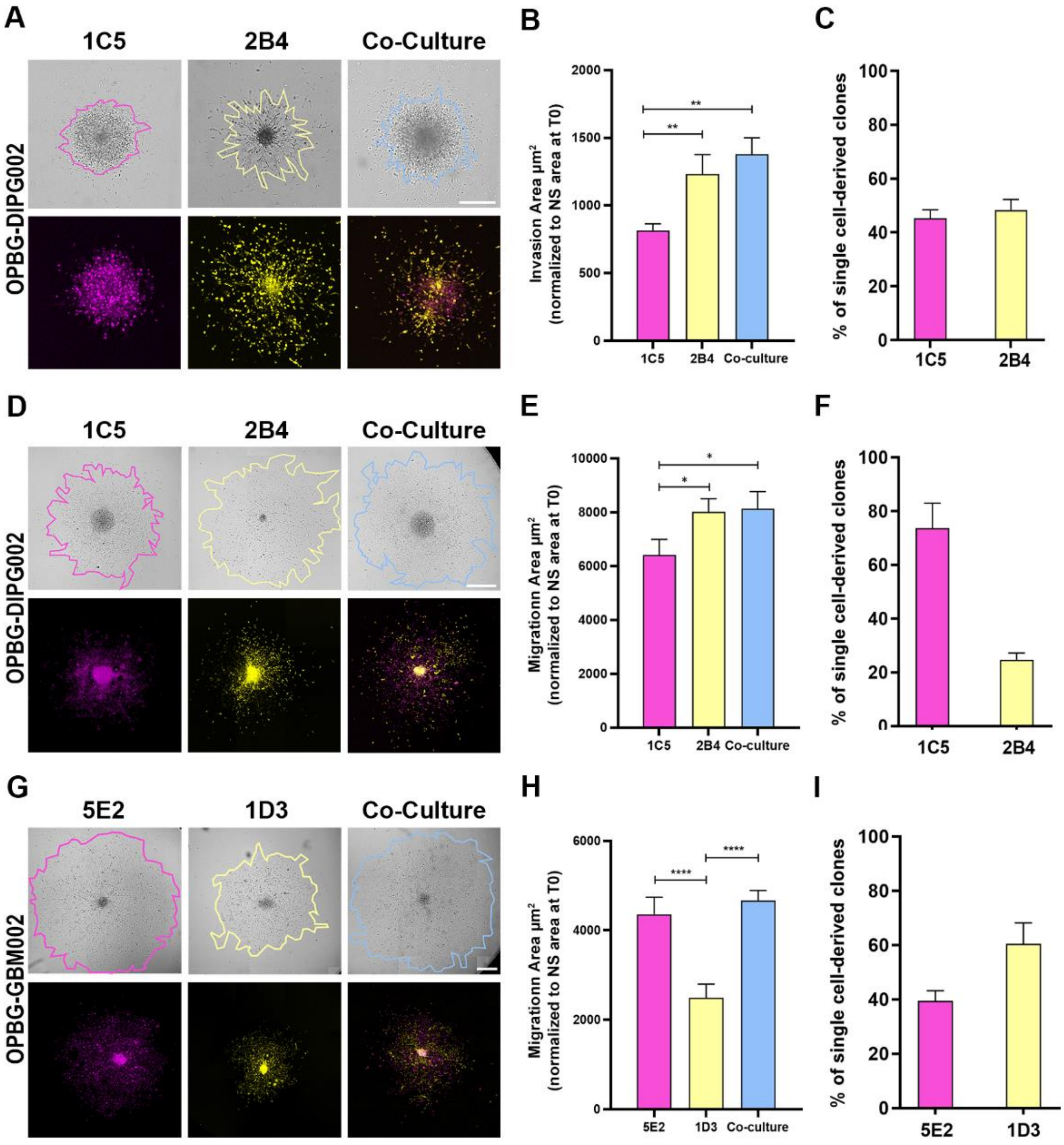

Supplemetary figure 3

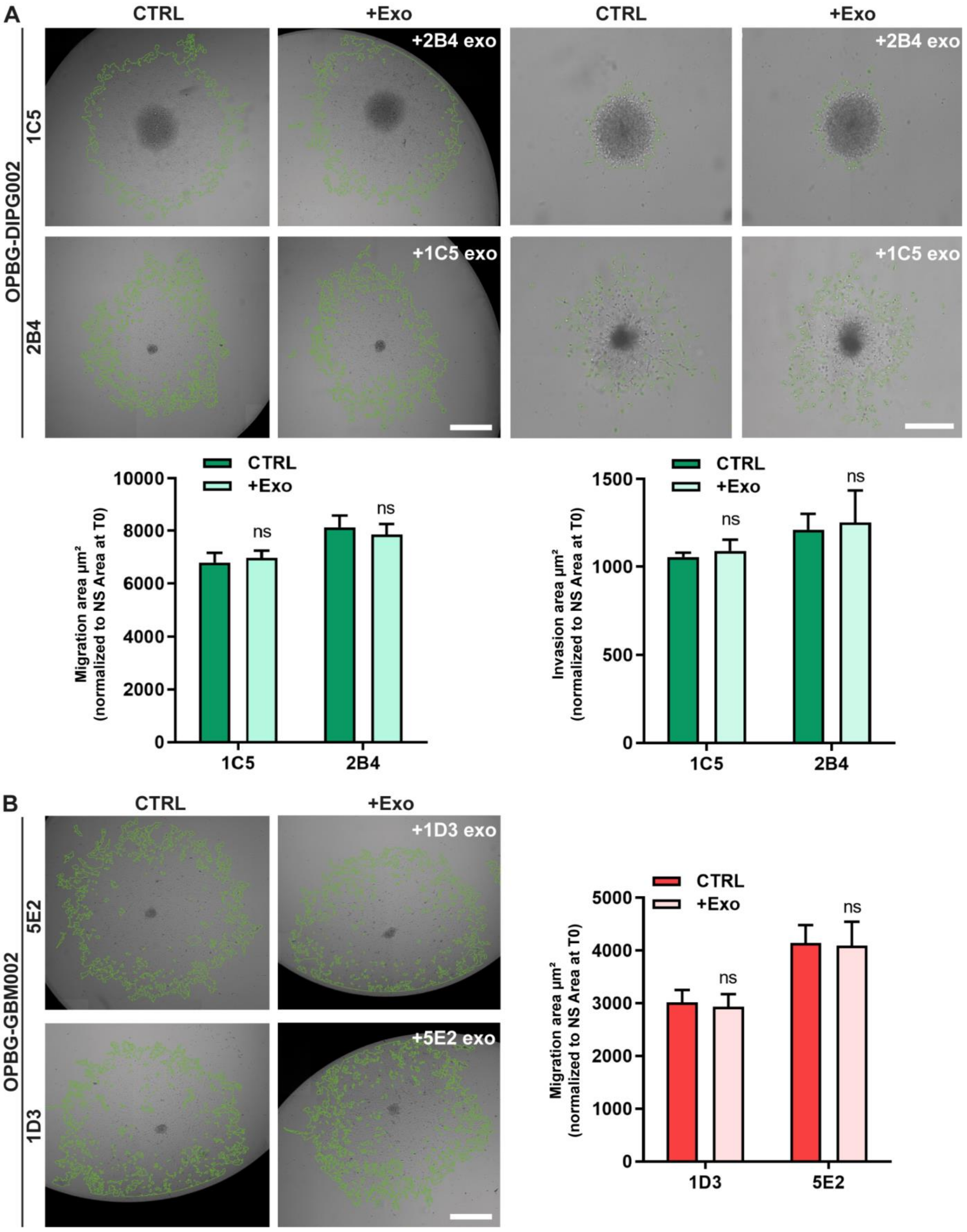

Supplementary figure 4

A

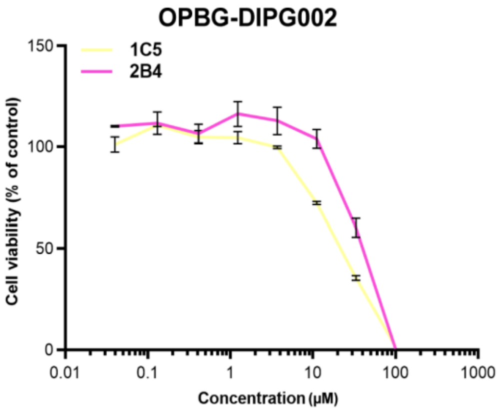

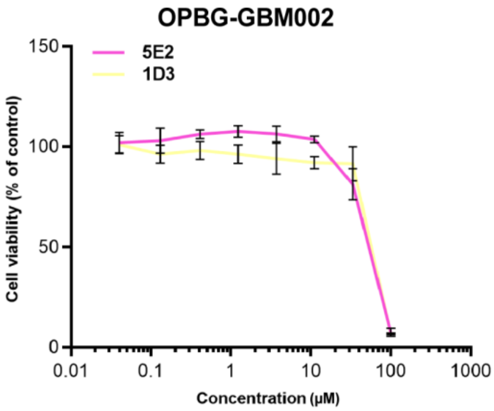

Cell lines	Clones	GI50
OPBG-DIPG002	1C5	24,6
	2B4	44,75
OPBG-GBM002	5E2	61,57
	1D3	66

Supplementary figure 5

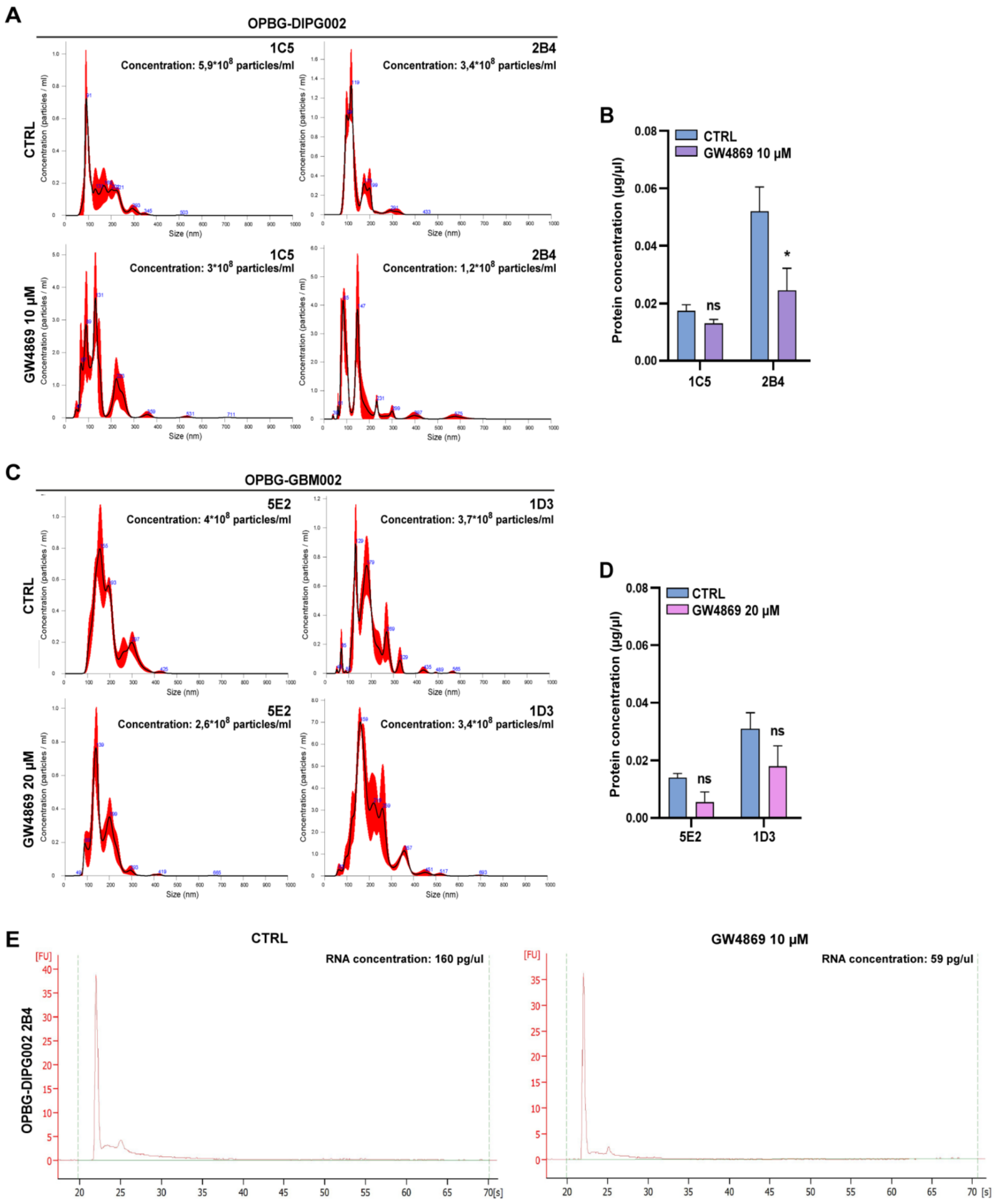

Supplementary figure 6

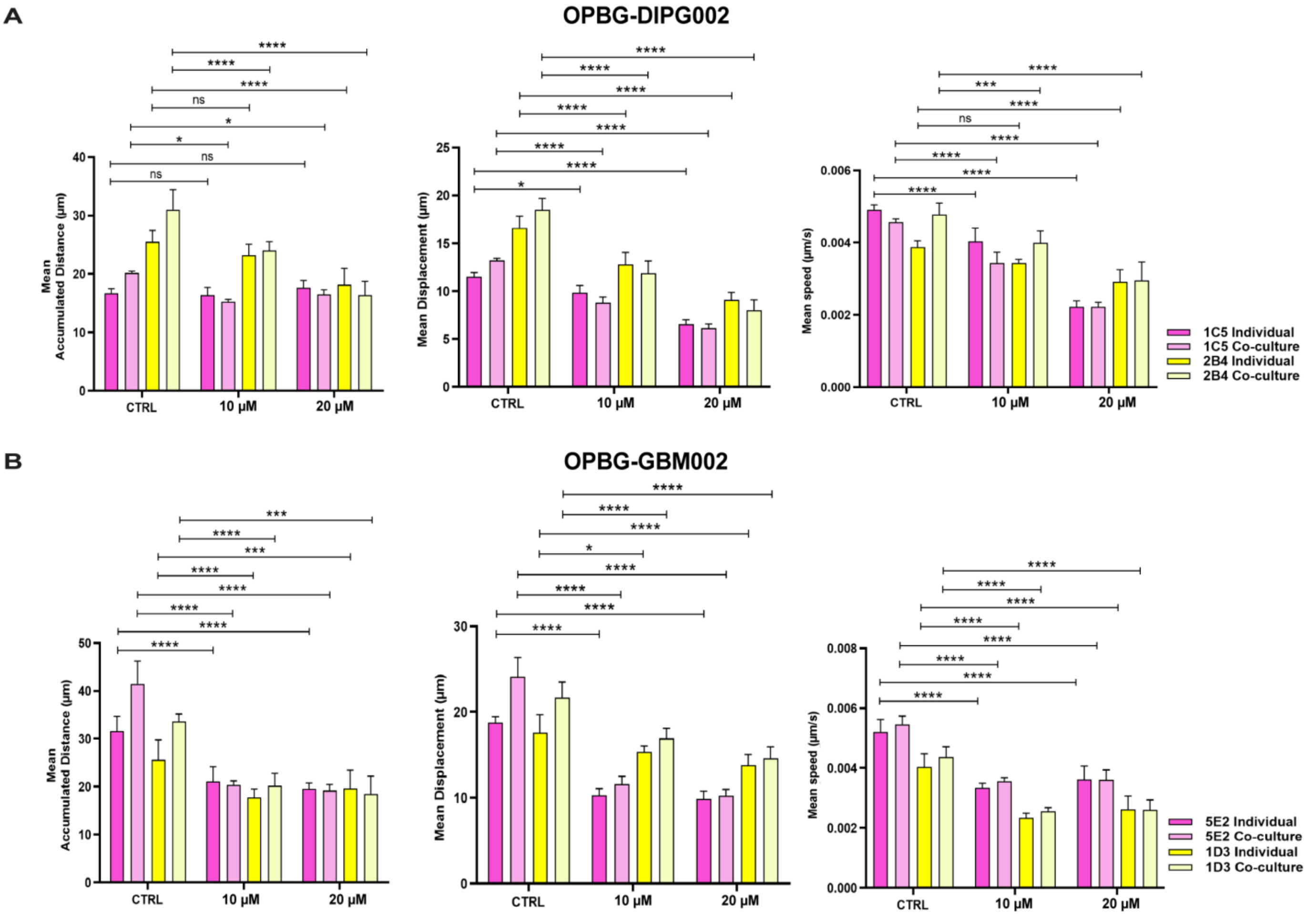

Supplementary figure 7

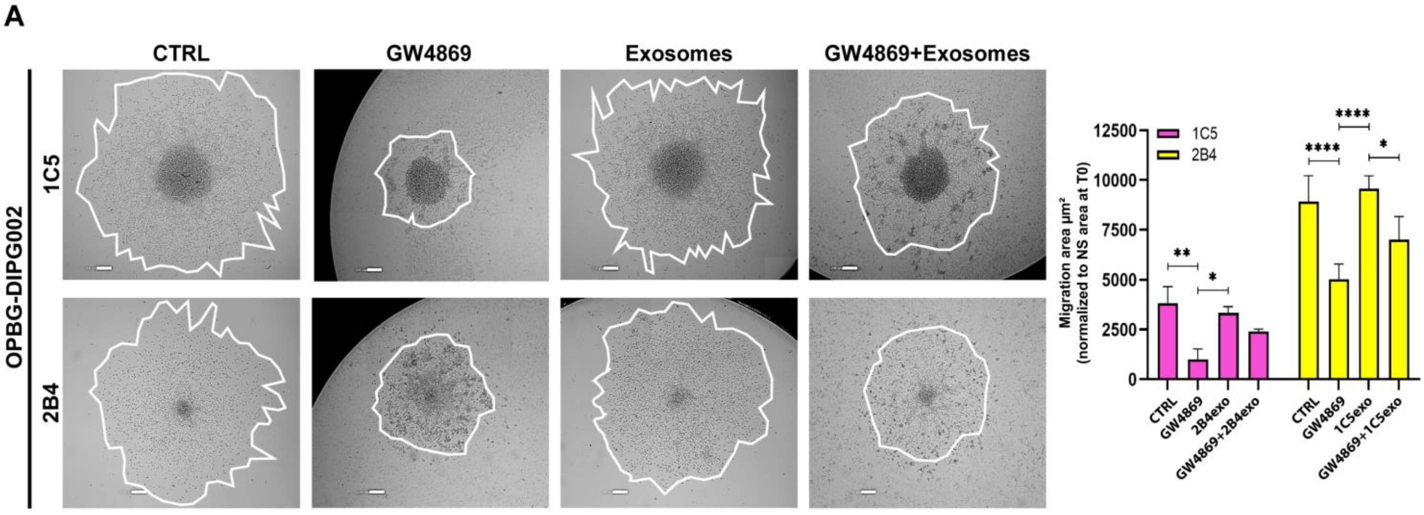

Supplementary figure 8

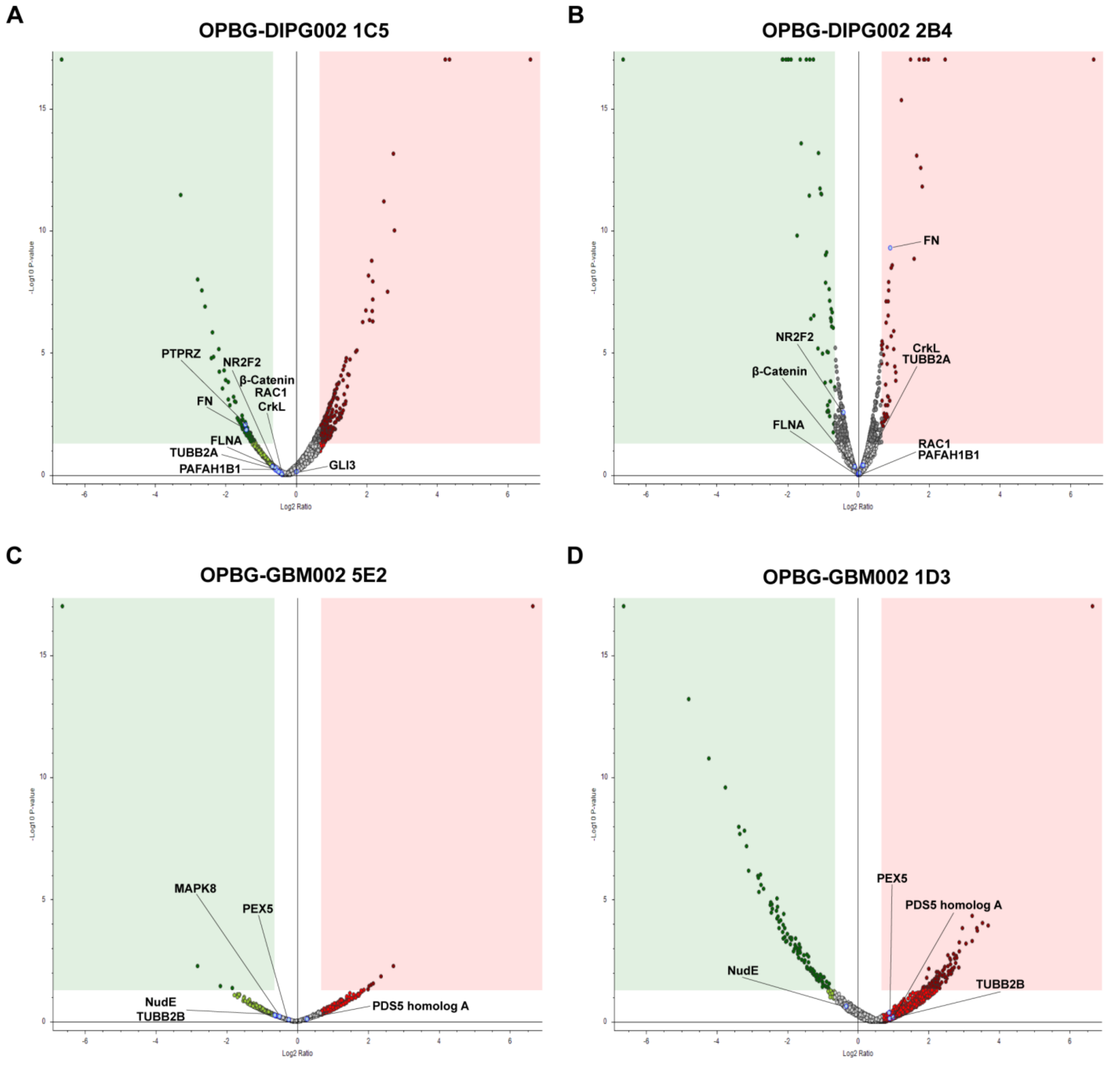

Supplement: Supplementary file 1 — Additional file 1: Figure S1. Overall Survival analysis of HGG patients using TCGA gene expression data. (A-B) The patient cohort was divided into two groups based on the median expression of OPBG-DIPG002 2B4 (A) and OPBG-GBM002 5E2 (B) specific gene signatures. The red curve represents patients with higher expression of this signature, while the blue curve represents patients with lower expression. Figure S2. Motility of single-cell-derived clones in mono and co-culture condition. Individual clones derived from OPBG-DIPG002 (A-D) and OPBG-GBM002 (G) were cultured either alone or co-cultured at an equal cell/cell ratio. Representative brightfield images are shown for the invasion (A) and migration (D-G) assays after 96 hours. The extent of invasion and migration is marked with coloured segmentation: co-culture (light blue); mono-cultures of 1C5 and 5E2 (fuchsia); mono-cultures of 2B4 and 1D3 (yellow). The total area covered by invading and migrating cells was quantified and normalised by the NS area determined at the time t0 (B-E-H). Scale bar = 500μm. Representative fluorescent images of OPBG-DIPG002 single-cell-derived clones 1C5 (Venus) and 2B4 (m-Orange2) 3D invasion (A) and migration (D) and of OPBG-GBM002 single-cell-derived clones 5E2 (Venus) and 1D3 (m-Orange2) migration (G) are shown as mono-culture and as co-culture (overlay of Venus and m-Orange2). Fluorescent images were acquired on a Leica TCS AOBS-SP8X confocal microscope. The percentage of each clone in the invasion (C) and migration (F-I) at 96 hours was analysed with Harmony software. Data are mean ± SD, n = 3. (****) p<0.0001; (***) p<0.001; (**) p<0.01; (*) p<0.05. Figure S3. The exosomal education of single-cell-derived clones does not affect their migratory/invasive phenotype. Exosomal education of the clones was performed. Exosomes isolated from donor clones were used to educate the recipient clone (10 μg/mL of media) every day, over 7 days. The effect on migration (A left panel and B) and inva [file 13578_2023_1166_MOESM1_ESM.pdf]
